# An update of the Worldwide Integrated Assessment (WIA) on systemic insecticides. Part 2: impacts on organisms and ecosystems

**DOI:** 10.1007/s11356-017-0341-3

**Published:** 2017-11-09

**Authors:** Lennard Pisa, Dave Goulson, En-Cheng Yang, David Gibbons, Francisco Sánchez-Bayo, Edward Mitchell, Alexandre Aebi, Jeroen van der Sluijs, Chris J. K. MacQuarrie, Chiara Giorio, Elizabeth Yim Long, Melanie McField, Maarten Bijleveld van Lexmond, Jean-Marc Bonmatin

**Affiliations:** 1 Utrecht University, Utrecht, The Netherlands; 2 School of Life Sciences, University of Sussex, Brighton, BN1 9QG UK; 3 Department of Entomology, National Taiwan University, Taipei, Taiwan; 4 RSPB Centre for Conservation of Science, The Lodge, Sandy, Bedfordshire SG19 2DL UK; 5 School of Life and Environmental Sciences, The University of Sydney, 1 Central Avenue, Eveleigh, NSW 2015 Australia; 6 Laboratory of Soil Biodiversity, University of Neuchâtel, Rue Emile-Argand 11, 2000 Neuchâtel, Switzerland; 7 Anthropology Institute, University of Neuchâtel, Rue Saint-Nicolas 4, 2000 Neuchâtel, Switzerland; 8 Centre for the Study of the Sciences and the Humanities, University of Bergen, Postboks 7805, 5020 Bergen, Norway; 9 Department of Chemistry, University of Bergen, Postboks 7805, 5020 Bergen, Norway; 10 Copernicus Institute of Sustainable Development, Environmental Sciences, Utrecht University, Heidelberglaan 2, 3584 CS Utrecht, The Netherlands; 11 Natural Resources Canada, Canadian Forest Service, 1219 Queen St. East, Sault Ste. Marie, ON P6A 2E5 Canada; 12 Aix Marseille Univ, CNRS, LCE, Marseille, France; 13 Department of Entomology, The Ohio State University, 1680 Madison Ave, Wooster, OH 44691 USA; 14 Smithsonian Institution, 701 Seaway Drive Fort Pierce, Florida, 34949 USA; 15 Task Force on Systemic Pesticides, Pertuis-du-Sault, 2000 Neuchâtel, Switzerland; 16 Centre National de la Recherche Scientifique (CNRS), Centre de Biophysique Moléculaire, Rue Charles Sadron, 45071 Orléans, France

**Keywords:** Systemic insecticides, Neonicotinoids, Fipronil, Insects, Pollinators, Soil biota, Aquatic organisms, Vertebrates, Ecosystem services, Review

## Abstract

New information on the lethal and sublethal effects of neonicotinoids and fipronil on organisms is presented in this review, complementing the previous Worldwide Integrated Assessment (WIA) in 2015. The high toxicity of these systemic insecticides to invertebrates has been confirmed and expanded to include more species and compounds. Most of the recent research has focused on bees and the sublethal and ecological impacts these insecticides have on pollinators. Toxic effects on other invertebrate taxa also covered predatory and parasitoid natural enemies and aquatic arthropods. Little new information has been gathered on soil organisms. The impact on marine and coastal ecosystems is still largely uncharted. The chronic lethality of neonicotinoids to insects and crustaceans, and the strengthened evidence that these chemicals also impair the immune system and reproduction, highlights the dangers of this particular insecticidal class (neonicotinoids and fipronil), with the potential to greatly decrease populations of arthropods in both terrestrial and aquatic environments. Sublethal effects on fish, reptiles, frogs, birds, and mammals are also reported, showing a better understanding of the mechanisms of toxicity of these insecticides in vertebrates and their deleterious impacts on growth, reproduction, and neurobehaviour of most of the species tested. This review concludes with a summary of impacts on the ecosystem services and functioning, particularly on pollination, soil biota, and aquatic invertebrate communities, thus reinforcing the previous WIA conclusions (van der Sluijs et al. 2015).

## Introduction

**Fig.1:**
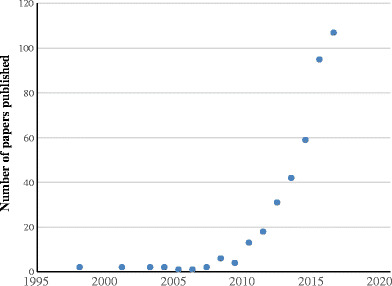
Number of research papers on pollinators and neonicotinoids published since 1998

Since the publication of the first Worldwide Integrated Assessment (WIA) review (Bijleveld van Lexmond et al. 2015) on the impact of neonicotinoids and fipronil systemic insecticides on invertebrates (Pisa et al. 2015), vertebrates (Gibbons et al. 2015), ecosystem services (Chagnon et al. 2015), and its conclusions (van der Sluijs et al. 2015), there has been a surge in publications related to this important issue. In particular, research on the impacts of these insecticides on bees and other pollinators has grown exponentially (Fig. 1) and IPBES published a review report on pollinators, pollination, and food production (IPBES 2016a), showing the great interest that this topic has raised worldwide. In this update, we have strived to collect all new information that has been published since 2014 onwards on the same topics covered by the WIA in 2015.

The first review paper of the updated WIA (Giorio et al. 2017, this special issue) deals with the mode of action of neonicotinoids and fipronil, their metabolism, synergies with other pesticides or stressors, degradation products, and the contamination of the environment by neonicotinoids and fipronil, including new insecticides introduced on the market.

For this second review paper, a broad-scaled literature search was performed using the Web of Science™ and Scopus® as reported by Gibbons et al. (2015) and restricted to the years 2014-early 2017. Search terms were [product] or “neonicotinoids,” and either “insects,” “invertebrates,” “vertebrates,” “mammals,” “birds,” “reptiles,” “amphibians,” “fish,” “soil biota,” “aquatic organisms,” and “ecosystem services,” where [product] was a placeholder for the name of each considered active ingredient (a.i.): imidacloprid, clothianidin, thiamethoxam, nitenpyram, acetamiprid, thiacloprid, dinotefuran, cycloxaprid, imidaclothiz, paichongding, sulfoxaflor, guadipyr, flupyradifurone, and fipronil. In addition, specific searches were made on a few common toxicity test species (e.g., rat) and by following up references cited in the publications found by the search. Therefore, the present review paper covers the effects on organisms, from aquatic and terrestrial invertebrates to vertebrates, and their impacts on ecosystems.

The updated WIA is divided in three parts, corresponding to effects on invertebrates (part A), vertebrates (part B), and ecosystems (part C).

Note that the third paper of the updated WIA discusses the efficacy of neonicotinoids and fipronil in agriculture and proposes some alternatives to the use of these products for pest control (Furlan et al. 2017, this special issue). It also summarizes the current regulations in Europe and other countries concerning these widely used systemic insecticides.

## Part A: invertebrates

### Effects of neonicotinoids and fipronil on pollinators

#### Honeybees (*Apis mellifera*)

Since the publication of the WIA document on the effects of neonicotinoid insecticides and fipronil on non-target invertebrates, research on this matter has continued. Lundin et al. (2015) provide a systematic review of research approaches, evaluating 268 publications on bees in general (honeybees, bumblebees, solitary bees). Another overview of scientific advances in the field of neonicotinoids and pollinators was made by Godfray et al. (2015). Van der Sluijs and Vaage (2016) reviewed the implications of the present pollinator crisis for global food security and concluded that it threatens global and local food security, can worsen the problems of hidden hunger, erodes ecosystem resilience, and can destabilize ecosystems that form our life support system. They call for an international treaty for global pollinator stewardship that simultaneously addresses its key drivers: creation and restoration of floral and nesting resources, a global phase out of prophylactic use of neonicotinoids and fipronil, improvement of test protocols in authorization of agrochemicals (see Sánchez-Bayo and Tennekes, 2017 for the changes that are needed), and restoration and maintenance of independence in regulatory science.

In the paragraphs below, results of recent studies with regard to honeybees (*Apis mellifera*) are listed, considering effects in vivo (field and semi-field situation) and in vitro (laboratory experiments).

### Field studies

Field studies to investigate effects of pesticides are observational in nature, making it hard to state causal relations between observed environmental variables and honeybee losses or honeybee health as these are dependent on a multitude of factors including weather, nutrition, genetics, pathogens and diseases, presence of multiple toxic compounds, potentially contrasting behavioral characteristics of the studied colonies, and very different methodological approaches.

Calatayud-Vernich et al. (2016) addressed this problem by using time series of counting dead bees in traps connected to hives in agricultural areas (Spanish citrus plantations), measuring the concentration of 58 different pesticides present in dead bees using LC-MS/MS. In this way, a change in mortality rate over time could be correlated to a differential presence of pesticides. The largest increases in mortality rate were associated with increased presence of dimethoate and chlorpyrifos. Imidacloprid was the fourth most present insecticide in dead bee samples, at concentrations varying between 12 and 223 ng/g dead bees. These concentrations are known to cause at least sublethal effects on bees (Decourtye et al. 2005), but increased exposure to imidacloprid presence could not be associated with bee mortality due to the presence of other pesticides. Kasiotis et al. (2014) used LC-ESI-MS/MS multiresidue analysis to investigate the presence and concentration of 115 pesticides in dead bees, pollen or honey collected by bees, focusing on pollen and honey collected by individuals or public authorities who evidenced specific high losses or bee death incidents in 2011, 2012, and 2013. Among the analyzed dead bees (*n* = 44), 50% were positive for clothianidin, 14% for chlorpyrifos, 9% for thiamethoxam, and 4.5% for imidacloprid. Concentrations of these compounds were mostly below the oral LD50 values for the compound detected, leading authors to state that more research is needed to determine the causal relations. However, the authors did not look at toxic metabolites of active compounds, possibly leading to an underestimation of compound presence.

An association between the presence of acetamiprid and thiacloprid in colonies (investigated by LC-MS/MS) and successive winter mortality was found by Van der Zee et al. (2015). In their observational study, the presence of these pesticides in any of the bee matrices (bees, pollen, wax and honey) was the second best predictor of winter loss in the observed population, the first being the amount of *Varroa destructor* in colonies in October. Their results indicate that the presence of acetamiprid and thiacloprid in honey is a better predictor of loss than its presence in bees and pollen. A similar field study by Budge et al. (2015) found a correlation between imidacloprid use in oilseed rape and colony mortality at the landscape level. Alburaki et al. (2015) monitored hives in neonicotinoid-treated corn areas and found elevated levels of acetylcholine esterase gene expression (a biomarker for physiological stress) in combination with higher pathogen and *Varroa* mite loads in hives from treated locations. In a later study, the same authors monitored colony performance and pesticide content of foragers and trapped pollen of colonies set up in neonicotinoid-treated corn fields and untreated corn fields (control) (Alburaki et al. 2017). They found no neonicotinoid compounds in foragers but sublethal amounts of thiamethoxam and clothianidin in trapped pollen. Mogren and Lundgren (2016), looking at pesticide presence in flowers seeded for pollinators adjacent to crop lands, found an association between presence of clothianidin and nutritional status of bees. Bees with increasing amounts of clothianidin had decreasing amounts of glycogen, lipids and protein.

Tsvetkov et al. (2017) measured long-term exposure (2 summers) to neonicotinoids in Canadian corn areas and matched their laboratory exposure parameters to this data. They found an association between field-realistic exposure to clothianidin and thiamethoxam and decreased colony immunity and survival. Moreover, both neonicotinoids became twice as toxic in the presence of field-realistic amounts of the fungicide boscalid. Using a large experimental design, Woodcock et al. (2017) allocated insecticide treatments (thiamethoxam, clothianidin, beta-cyfluthrin, lambda-cyhalothrin), fungicide treatments (thriam, prochloraz, fludioxonil, metalaxyl-M) and standardized colonies of *Apis mellifera*, *Bombus terrestris*, and units of *Osmia bicornis* to a total of 33 sites with oilseed rape in the UK, Hungary, and Germany. They found partly significant negative effects on honeybee worker numbers and egg laying in the UK and Hungary, but not in Germany. Their results suggest interaction effects of treatment with the environment, available flora, and residues of earlier treatments not part of the experiment. It should also be noted that all treatments including controls also received fungicide treatments and that different fungicides were used in the three different countries. Rolke et al. (2016) carried out a large field study of the effects of clothianidin-dressed oilseed rape on honey bees, finding no adverse effect of treatment on numbers of adult bees or brood, although the study had no replication (only one treated and one control site) and therefore these results should be accorded little weight.

Wegener et al. (2016) have measured 28 biochemical, biometrical, and behavioral aspects of honeybees (*A. mellifera*) to investigate the effect of imidacloprid and fenoxycarb on colony productivity and survival. Imidacloprid affected honey yield, total number of bees, and the activity of the enzyme phenoloxidase in worker bees.

Pilling et al. (2013) exposed hives to corn and oilseed rape plots treated with thiamethoxam and found no effects on colony parameters (mortality, colony strength, amount of brood and honey). However, this study co-published by the manufacturer (Syngenta Ltd) was criticized by Hoppe et al. (2015), who pointed to several weaknesses: the use of a non-commercial pesticide formulation, lower than field-realistic doses, flawed experimental design, and lack of statistical analysis. The latter was also subject to criticism by Schick et al. (2017) who pointed to the low quality of the data and consequent lack of power to estimate effects.

Garbuzov et al. (2015) added to the discourse on honeybee field studies with their findings that oil seed rape, a potential exposure crop to neonicotinoid pesticides, elicited less foraging then expected by its presence in the landscape.

An example of the requirements of large field studies and their implementation is given by Heimbach et al. (2016). A wider review of neonicotinoid field studies by the industry can be found in Schmuck and Lewis (2016). Bakker (2016) points at shortcomings in the current field study protocols as used by the European Food Safety Authority (EFSA) and proposes ideas to disentangle effect measurements of acute and sublethal effects in experiments. Improvement of research methods (sampling and measuring exposure) is also addressed by Benuszak et al. (2017). Hesketh et al. (2016) provide arguments for an increased exposure time (> 240 h) to better identify sublethal effects in honeybee toxicity test, the current standard being 96 h of exposure.

A modeling approach for sublethal effects of pesticides on colony level, using the BEEHAVE model, can be found in Thorbek et al. (2017a). Their model study suggests that monitoring of field experiments must continue for at least 1 month to identify sublethal effects. In another publication the authors criticize the Khoury bee population model used by EFSA to set exposure values for colony losses related to pesticides as too conservative (Thorbek et al. 2017b).

An interesting study that tries to bridge the gap between field and laboratory studies has been done by Henry et al. (2015). In their study, they show that a mixture of effects on individual bees leads to demographic effects in the colony and can lead to negative outcomes at the population level.

Reports on pesticide presence in dead bees generated by investigating reported colony losses by monitoring agencies give information about the variety and quantity of pesticides used in the bees’ environment. A review on recent acute bee poisonings, with a focus on Eastern Europe, is given by Kiljanek et al. (2017). Kiljanek et al. (2016) and Kimura et al. (2014) provide information of poison incidents in a Japanese region. A recent study in France (Daniele et al. 2017) has shown that neonicotinoids and boscalid were the most detected pesticides in honeybees, beebread, and wax, for numerous samples primarily taken from symptomatic colonies during springs 2012–2016.

As stressed in the introduction to this section, observational studies do not suffice to demonstrating causality and other pesticides or other environmental factors may be involved in the observed responses. However, an increasing number of field studies include physiological and behavioral analyses (linking field- to controlled laboratory studies) that allow more causal interpretations of the impact of neonicotinoids on bees. These studies converge in clearly demonstrating the existence of a significant detrimental impact on bees.

### Semi-field studies

Sandrock et al. (2014b) used a fully crossed experimental design (sister queens, in-hive pollen feeding) to test effects of clothianidin (2 ppb in pollen) and thiamethoxam (5 ppb in pollen) administered during two brood cycles on colony performance and queen supercedure. They found that the number of bees and brood rearing decreased and queen supercedure increased in treated colonies. After winter, treated colonies exhibited a lower swarming tendency, possibly related to their lower growth rate. Interestingly, they found a difference in effect for the 2 races of honeybees they used (*A. m. mellifera* and *A. m. carnica*), with bees originating from an area with intensive agriculture including pesticide application (*A. m. carnica*) experiencing less effects of the treatments than bees from a more natural habitat (*A. m. mellifera*), possibly pointing toward a genetic adaptation. Though not a semi-field study, the results of Rinkevich et al. (2015) also indicate dramatic differences between races in sensitivity to neonicotinoids.

In a semi-field study by Henry et al. (2015) thiamethoxam-coated oilseed rape was sown in a specific study area (total of 288 ha in 2 years) and hives were placed at various distances and directions to generate a range of exposure levels. Monitoring of colony demographics showed that more exposed colonies had a greater loss of forager bees, but the numbers of foragers were buffered by colony regulation response. However, the effects of population changes within the beehive (larvae, nurses, workers, foragers) could weaken the colony. Dively et al. (2015) conducted a 3-year study feeding pollen supplements laced with imidacloprid (5, 20, and 100 μg/kg). They found an association between higher doses (20 and 100 μg/kg) and reduced winter survival. Higher dose colonies also had a higher *Varroa* mite load. Exposure to imidacloprid and clothianidin lead to colony collapse symptoms at the end of winter in half of the small study population used by Lu et al. (2014).

Tison et al. (2016) used harmonic radar to track bees at feeders spiked with low doses of thiacloprid and unspiked controls. They found that foraging life of bees using the spiked feeder was shorter and that exposed bees made more navigation errors, had less homing success, and showed impaired social communication.

Stanley et al. (2015b) tested a range of pesticides, including acetamiprid, imidacloprid, and thiamethoxam in both laboratory assays (topical application and filter paper contact) and in semi-field settings (pesticides applied to potted plants moved to field, application of pesticides in field directly) for toxicity and repellent effects, using both *Apis mellifera* and *Apis cerana*. The neonicotinoids had less direct toxicity (less lethal) than for example deltamethrin and malathion but big differences were found between topical, filter paper, and field applications for several tested substances.

### Experimental (in vitro) studies

In comparison to field and semi-field studies, where it is extremely difficult to control unwanted (and unknown) influences, properly conducted experimental assays allow for causal arguments about exposure-effect relationships. Exposure of bees to pesticides is most often done by feeding bees with known amounts of the active substance and measuring the lethal or sublethal responses. Whereas lethality is easily observed, sublethal effects can vary greatly in their way of occurrence (including cascade effects) and intensity in a honeybee colony.

#### Sublethal effects on memory, behavior, and locomotion

Karahan et al. (2015) found that feeding honey bees with field-realistic doses of imidacloprid (0.36 to 7.20 ng/bee) negatively affected the number of foraging trips, number of foragers returning, and flowers visited. Roat et al. (2014) found changes in the brain proteome of Africanized honeybees for doses of 10 pg fipronil per day during 5 days. Concentrations of several brain proteins involved in detoxification, glycolysis, and cell growth were altered, possibly leading to memory and learning impairment and to a reduced life span. Zaluski et al. (2015) also used Africanized honeybees in their study about effects of fipronil on colony development and bee motoric control and behavior. Treated adult bees (1/500th of the LD50) bees showed reduced motor activity and became lethargic, while treated colonies showed a reduction in egg laying and larval numbers.

Tan et al. (2015) investigated the effect of imidacloprid on adult bee memory and learning behavior by feeding total doses of 0.24 ng to larvae of *A. cerana*. They found that long-term memory, but not short-term or larval survival, was affected by the treatment. Also using *Apis cerana* in an earlier study, these authors found that trained exposed bees foraged less and had a lower avoidance of predators (i.e., Asian hornet *Vespa velutina*) (Tan et al. 2014). Wright et al. (2015) used a choice assay with imidacloprid and thiamethoxam influencing olfactory memory. They found that low acute doses affect olfactory memory negatively, with this effect being greater than the effect on memory. Doses of imidacloprid (11.25 ng/bee), clothianidin (2.5 ng/bee), and thiacloprid (1.25 mg/bee) given to trained forager bees resulted in less successful returns and a lower ability to navigate (Fischer et al. 2014). Effects on learning and memory were also found by Mengoni Goñalons and Farina (2015) who fed sublethal doses of imidacloprid to young bees. They postulate that impaired memory and sensitivity to rewards of individual bees affects colony performance.

Peng and Yang (2016) found a reduction of mushroom bodies in parts of the brain responsible for olfactory and visual processing. At the molecular level, interaction between odor binding proteins and imidacloprid has been studied in *A. cerana* by Li et al. (2015a), who found that presence of imidacloprid decreased the affinity of a specific odor binding protein and a flower volatile.

A 24-h exposure of adult bees to imidacloprid, dinotefuran, clothianidin, and thiamethoxam at sublethal field-realistic doses (0.323 to 0.481 ng/bee) resulted in behavioral changes. Bees walked less and groomed more (Williamson et al. 2014). Blanken et al. (2015) used flight cages to determine effects of imidacloprid (about 6 ng/mL, weekly feeding of 660 mL in a 13-week period) on flight capacity of forager bees, in combination with differential *Varroa* destructor mite loads of the bee donor colonies. Their results showed an interaction between physiological stressed caused by *Varroa* and imidacloprid, with imidacloprid possibly affecting the body mass of bees and lower body mass causing decreased flight capacity. An interesting finding is that of Kessler et al. (2015). Their data generated by choice assays (sucrose laced with imidacloprid or thiamethoxam versus plain sucrose) suggest that bees prefer solutions with imidacloprid and thiamethoxam. Another study investigating effects on food consumption found that thiamethoxam decreased bees’ response to higher sucrose concentrations (Démares et al. 2016). Alkassab and Kirchner (2016) exposed winter bees to sublethal doses of clothianidin and measured behavioral effects. Chronic exposure to 15 ppb was found to significantly affect long-term memory. Both deltamethrin and acetamiprid were used in retrieval assays (conditional proboscis response) by Thany et al. (2015). Their results showed that retrieval was impaired at lower doses of acetamiprid compared to deltamethrin.

Papach et al. (2017) present the first evidence of impaired learning and memory in adult bees that were fed thiamethoxam (0.6 ng/bee) during the larval stage. Colony survival critically depends on successful learning and memory. Chronic larval exposure to sublethal doses of this neonicotinoid resulted in alterations of associative behavior in adults. Similar delayed effects on learning and memory following larval exposure have been reported for other neonicotinoids such as imidacloprid (these studies are reported in the WIA 2015 study).

Effects of sublethal doses of thiacloprid on social interactions and network structure established by a group of honeybee worker individuals has been quantified in a study by Forfert and Moritz (2017) using experimental groups. Bees fed with thiacloprid (0.17 and 0.80 μg thiacloprid in 20 μL 2.7 M sucrose solution) significantly reduced their network centrality, but they nevertheless exchanged more food to other group members, which resulted in a dilution of the contaminated food. The authors argue that although thiacloprid may act as a general perturbator of social network structure, it still may play a role in the dynamics of disease transmission in the colony if pathogens are transmitted via food exchange.

Using flight mills, Tosi et al. (2017) found that flight activity (duration and distance) was increased after a single sublethal dose and decreased (duration, distance, velocity) after 1–2 days of chronic exposure.

To understand how neonicotinoids affect behavior and immunity at the molecular level, Christen et al. (2016) looked at transcriptional regulation of 8 genes in caged honeybees fed with field-realistic concentrations of acetamiprid, clothianidin, imidacloprid, and thiamethoxam. They found downregulation of transcription of two genes involved in memory and increased transcription of the gene responsible for vitellogenin, the latter possibly affecting foraging behavior. A follow-up study confirmed these results and looking at effects of binary mixtures of acetamiprid, clothianidin, imidacloprid, and thiamethoxam on memory and vitellogenin gene transcription, found smaller effects of mixtures opposed to single substance application on gene regulation (Christen et al. 2017).

#### Sublethal effects on immunity and metabolism

Gene expression profiles in honeybee midgut showed that insecticide treatments (imidacloprid or fipronil) had no impact on detoxifying genes but led to a significant downregulation of immunity-related genes, suggesting a possible immunotoxicity of neonicotinoid and phenylpyrazole insecticides under chronic exposure (Aufauvre et al. 2014). This study also showed that *N. ceranae* + fipronil and *N. ceranae* + imidacloprid combinations do not systematically lead to a synergistic effect on honeybee mortality. Brandt et al. (2016) found that imidacloprid, thiacloprid, and clothianidin caused reduced hemocyte density, encapsulation response, and antimicrobial activity after a relatively short exposure (24 h) to field-realistic concentrations. Looking specifically at the interaction of thiacloprid and the pathogens *Nosema ceranae* and black queen cell virus, Doublet et al. (2014) found that thiacloprid increased the viral load of larvae and so negatively affected larval survival, as well as aggravating the effect of *Nosema* on adult mortality. A similar study by Gregorc et al. (2016) combined exposure to *Nosema ceranae* and thiamethoxam and showed no synergistic effects of the two. Reviews of the relation between nicotinoid pesticides and honeybee disease can be found in Sánchez-Bayo and Desneux (2015) and Sánchez-Bayo et al. (2016b).

Badawy et al. (2015) measured the effects of oral and topical application of four pesticides (acetamiprid, dinotefuran, pymetrozine, pyridalyl) on detoxifying enzyme activity (acetylcholinesterase, carboxylesterase, glutathione-S-transferase and polyphenol oxidase). They found dinotefuran to be the most toxic, pyridalyl second and acetamiprid/pymetrozine the least toxic. Carboxylesterase and glutathione-S-transferase were able to detoxify low doses of acetamiprid, pymetrozine, and pyridalyl but not dinotefuran. Böhme et al. (2017) were feeding pollen containing mixtures of pesticides at field-realistic (sublethal) doses to determine synergistic effects, as exposure to multiple substances through pollen is common but little studied. They found that larval weight was higher and acini diameters of the hypopharyngeal glands of nurse bees were smaller in the experimental group. Renzi et al. (2016) also looked at hypopharyngeal glands and found that dietary exposure to thiamethoxam was associated with smaller acini and lower total protein content of bee heads.

Exposure to thiamethoxam was also found to alter thermoregulation in individual bees, with effects dependent on ambient temperature and dose (Tosi et al. 2016). At higher temperatures (33 °C), body temperature of exposed bees increased, whereas lower temperatures (22 °C) lead to lower body temperature 60–90 min post treatment. In both exposed groups, body temperatures were lower than control group the following day.

An interesting finding was done by Rittschof et al. (2015), who investigated aggressive behavior of honeybees as a result of early-life social experience, using acetamiprid as a stressor to identify effects on the immune system. Their results found that aggressive bees had less immunosuppressive effects of acetamiprid than less aggressive bees.

#### Sublethal effects on reproduction

Sublethal effects on honeybee reproduction were not mentioned in the original WIA article on invertebrates (Pisa et al. 2015) but might be of considerable importance, as specific effects on, for example, sperm viability and queen mating success might directly affect population numbers. Williams et al. (2015) found that queens exposed to clothianidin and thiamethoxam had larger ovaries and reduced quality and quantity of sperm stored in the spermatheca. Very low doses of imidacloprid, alone and in combination with the parasite *Nosema ceranae*, were found to increase activity of detoxifying enzymes and decrease survival of queens (Dussaubat et al. 2016).

Drones that were raised in semi-field and laboratory conditions and exposed to fipronil through feeding showed a decrease in quantity of spermatozoa and increased mortality of spermatozoa (Kairo et al. 2017). This confirmed earlier research by the same authors had shown that queens inseminated with sperm of fipronil exposed drones had less and less viable spermatozoa stored in their spermatheca (Kairo et al. 2016). They found that several pesticides, among them fipronil, imidacloprid, and thiamethoxam, reduced sperm viability (in vitro sperm assay). Effects on drones were also found by Straub et al. (2016), who reported reduced drone life span as well as decreased sperm quality (low quantity of spermatozoa, reduced viability by 40%). Number of newly emerged adults and drone body mass was unaffected. Sublethal dose of imidacloprid (2 ppb) decreased also sperm viability by 50% 7 days after treatment in another study (Chaimanee et al. 2016).

Wu-Smart and Spivak (2016) fed small (1500–7000 bees) colonies with different doses of imidacloprid (0, 10, 20, 50, and 200 ppb) in syrup for 3 weeks to investigate its effect on queen productivity. They observed a decrease in egg laying rate and queen motility associated with exposure, as well as negative effects on foraging, hygienic behavior of worker bees, and on colony development in all treated colonies. Independent of colony size, number of eggs laid per 15 min was reduced by approximately 50% by 10 ppb imidacloprid compared to control. These findings demonstrate that chemical exposure may affect sperm quality in the spermatheca of honey bee queens, queen fecundity, threatening the reproductive success and survival of the colony.

An interesting study on honeybee reproductive metabolism was done by Wessler et al. (2016). They looked at the effect of thiacloprid and clothianidin on the secretion of acetylcholine by the hypopharyngeal gland. Acetylcholine is a key compound of larval food and royal jelly. Release of acetylcholine and its presence in larval food decreased by 80% after 4 weeks of exposure to high doses of both neonicotinoids. Field-realistic doses (200 ppb for thiacloprid, 1 to 10 ppb for clothianidin) lowered acetylcholine in larval food and showed negative effects on brood development.

#### Sublethal effects due to ontogenic exposure

Residue analyses of pollen, honey or bee wax revealed the presence of a cocktail of multiple insecticides accumulating at the same time (Bonmatin et al. 2015; David et al. 2016; Krupke and Long 2015; Mullin et al. 2010; Daniele et al. 2017; Giorio et al. 2017 this special issue). However, relatively few investigations have focused on the sublethal effects of pesticides on the honeybee brood.

It has been clearly shown that rearing brood in contaminated combs causes delayed development of larvae and emergence as well as a shortened adult life span (Wu et al. 2011). An additive interaction between black queen cell virus (BQCV) and thiacloprid on host larval survival was also observed (Doublet et al. 2014). A recent study by López et al. (2017) demonstrated a synergistic interaction when larvae are exposed to sublethal doses of dimethoate or clothianidin in combination with *Paenibacillus larvae*, the causative agent of American foulbrood (AFB). It is evident that the cellular response of larvae to individual and combined stressors allows for unmasking previously undetected sublethal effects of pesticides on colony health (Giorio et al. 2017, this special issue).

Bee larvae that were fed sublethal doses of thiamethoxam by Tavares et al. (2015a) showed condensed cells and early cell death in the optical lobe part of the brain, as well as dose-dependent effects on development speed and body size.

By exposing a hive to imidacloprid, Yang et al. (2012) discovered that honey bee larvae fed with a sublethal dose of imidacloprid still completed their development into adult bees, but they did so with a decreased olfactory learning ability. This impairment occurred with a dose that could be as little as 0.04 ng per larva. These results demonstrate that sublethal dosages of imidacloprid given to the larvae affect the subsequent associative ability of the adult honeybee workers. Peng and Yang (2016) further revealed the effect of sublethal doses of imidacloprid on the neural development of the honeybee brain by immune-labeling synaptic units in the calyces of mushroom bodies. This not only links a decrease in olfactory learning ability to abnormal neural connectivity but also provides evidence that imidacloprid damages the development of the nervous system in regions responsible for both olfaction and vision during the larval stage of the honeybee.

To reveal the potential spectrum of sublethal effects of imidacloprid exposure in the larval stage, Wu et al. (2017) measured changes in global gene expression in the heads of newly emerged adults. They found that multiple physiological changes could be induced by the sublethal exposure to imidacloprid, affecting detoxification, immunity, sensory processing, neuron development, metabolism, mitochondria, and synthesis of royal jelly.

### Other pollinators

#### Direct lethality of neonicotinoids to wild bees

Around 2000 bee species are known from Europe, with 400 of these classified as endemic (Nieto et al. 2014). The biology, behavior, and ecology of each of these species differ from those of honeybees, for example, some bees ingest pollen for transport (e.g., *Hylaeus* sp.), which might provide much greater exposure than carrying pollen in corbiculae. Consequently, extrapolating from the limited toxicological data available for 19 bee species to the effects of neonicotinoids on the wider European fauna is fraught with difficulties given the wide variation in relative sensitivity, ecology, and behavioral traits. Conversely to the results of Cresswell et al. (2012) who exposed bumble bees and honey bees to high doses, current data suggests that wild bees are equally to slightly less sensitive to neonicotinoids compared to honeybees when considering direct mortality (e.g., Sánchez-Bayo et al. 2017). However, care must be taken when considering individual bee species, genera, and families, as different taxonomic groups may show consistently different individual-level sensitivity. Most European wild bees are smaller than honeybees and there is the potential for them to be more sensitive on a basis of a few ng/bee exposure. In general, continuing to use honeybee neonicotinoid sensitivity metrics is likely to be a reasonable proxy measure for the direct sensitivity of the wild bee community to neonicotinoids (Arena and Sgolastra 2014), but further work is needed in this area to cover the wide range of bee species present in agricultural environments.

In large parts of Asia, the ecological niche of *Apis mellifera* is occupied by the similar but distinct sister species *A. cerana*. As agriculture has intensified and pesticide use increased strongly, effects can be expected on this bee species but little toxicological research has been conducted so far. The study of Yasuda et al. (2017) addresses this knowledge gap. They used the subspecies *A. cerana japonica* to determine LD50 values for acute contact toxicity for commonly used pesticides. Of the neonicotinoid group, dinotefuran proved to be most toxic (1.4 ng/bee), followed by thiamethoxam (2.4 ng/bee), clothianidin (3.4 ng/bee), imidacloprid (3.6 ng/bee), and acetamiprid (278 ng/bee). This LD50 for fipronil was determined at 2.5 ng/bee. The authors note that *A. cerana* is generally more sensitive to pesticides and that results obtained for *A. mellifera* cannot be generalized to *A. cerana*.

Arena and Sgolastra (2014) conducted a meta-analysis comparing the sensitivity of wild bees to pesticides relative to the sensitivity of honeybees. This analysis combined data from 47 studies covering 53 pesticides from six chemical families with a total of 150 case studies covering 18 bee species (plus *A. mellifera*). The authors calculated a sensitivity ratio (*R*) between the lethal dose for species a (*A. mellifera*) and for species s (other than *A. mellifera*), where *R* = LD50a/LD50s. A ratio of over 1 indicates that the other bee species is more sensitive to the selected pesticides than *A. mellifera* and vice versa. There was high variability in relative sensitivity ranging from 0.001 to 2085.7, but across all pesticides a median sensitivity of 0.57 was calculated, suggesting that *A. mellifera* was generally about two times more sensitive to pesticides than other bee species. In the vast majority of cases (95%), the sensitivity ratio was below 10.

Combining data for all neonicotinoids (acetamiprid, imidacloprid, thiacloprid, and thiamethoxam) and for both acute contact and acute oral toxicity, nine studies covering nine bee species (plus *A. mellifera*) were found. These studies showed a median sensitivity ratio of 1.045 which is the highest median value of all the analyzed pesticide chemical families. The most relatively toxic neonicotinoids to other bees were the cyano-substituted neonicotinoids acetamiprid and thiacloprid as these pesticides exhibit lower toxicity to honeybees than the nitro-substituted neonicotinoids imidacloprid and thiamethoxam.

In 2013, the EU installed a partial ban on imidacloprid, clothianidin, thiamethoxam, and fipronil while allowing continued use of acetamiprid and thiacloprid. Searching for studies about effects of the banned compounds including both acute contact and acute oral toxicity, 12 studies covering 10 bee species (plus *A. mellifera*) were found. These studies showed a median sensitivity ratio of 0.957 which is close to the calculated sensitivity ratio for all neonicotinoids. Studies on *Bombus terrestris* consistently report a lower sensitivity ratio between 0.005 and 0.914, median 0.264. *Bombus terrestris* is widespread in Europe and is the most commonly used non-Apis model system for assessing the effects of neonicotinoids on wild bees. Differences in bee body weight have been proposed to explain these differences, with sensitivity to pesticides inversely correlated with body size (Devillers et al. 2003). However, this has not been consistently demonstrated and other mechanisms have been suggested such as species-level adaptation to feeding on alkaloid-rich nectar (Cresswell et al. 2012). With the limited data available, Arena and Sgolastra (2014) could not comment on the strength of these claims and further experiments are needed.

Spurgeon et al. (2016) calculated various toxicity measures of clothianidin on honeybees, the bumblebee species *B. terrestris* and the solitary bee species *Osmia bicornis*. Acute oral toxicity 48, 96, and 240 h LD50s for honeybees were 14.6, 15.4, and 11.7 ng/bee, respectively. For *B. terrestris*, the corresponding values were 26.6, 35, and 57.4 ng/bee, respectively. For *O. bicornis*, the corresponding values were 8.4, 12.4, and 28.0 ng/bee, respectively. These findings are generally in line with the findings of Arena and Sgolastra (2014), with *B. terrestris* less sensitive than *A. mellifera* at all time points and *O. bicornis* less sensitive at 240 h.

Sgolastra et al. (2017) calculated relative sensitivity to clothianidin to these same three species over a range of time periods from 24 to 96 h. The highest LD50 values were obtained after 24 h for *A. mellifera* and *B. terrestris* and after 72 h for *O. bicornis*. At these time points, *O. bicornis* was the most sensitive of the three species, with LD50 measurements of 1.17 ng/bee and 9.47 ng/g, compared to 1.68 ng/bee and 19.08 ng/g for *A. mellifera* and 3.12 ng/bee and 11.90 ng/g for *B. terrestris*. These results are in line with the values calculated by Spurgeon et al. (except for the 240 h values), with decreasing sensitivity in the order of *O. bicornis* > *A. mellifera* > *B. terrestris*. Together, these studies support the position that small bodied species show greater sensitivity to neonicotinoids.

Czerwinski and Sadd (2017) found detrimental interactions of imidacloprid exposure and bumblebee immunity. Adult workers of *Bombus impatiens* received 6-day pulses of either low (0.7 ppb) or high (7 ppb) field-realistic doses of imidacloprid. This was followed by an assay to test immunity and survival following a nonpathogenic immune challenge. The results showed that high-dose imidacloprid exposure reduces constitutive levels of phenoloxidase, an enzyme involved in melanization. Hemolymph antimicrobial activity initially increases in all groups following an immune challenge, but while heightened activity is maintained in unexposed and low imidacloprid dose groups, it is not maintained in the high exposure dose bees, although exposure had ceased 6 days prior. When imidacloprid exposure was followed by an immune challenge, a significantly decreased in survival probability was observed relative to control bees and those only immune challenged or imidacloprid exposed. A temporal lag for immune modulation and combinatorial effects on survival suggest that resource-based trade-offs may, in part, contribute to the detrimental interactions. These findings are particularly relevant because such impairment of the immune system at field-realistic exposure to neonicotinoids is likely to have health consequences for pollinators that in real life often face multiple stresses of sublethal neonicotinoid exposure and pathogens. It also raises a broader question whether impairment of the immune system by neonicotinoids is limited to insects or whether it also affects other non-target species that are exposed.

Baron et al. (2017) provides the first evidence of impacts of thiamethoxam on the ovary development and feeding of spring-caught wild queens of four bumblebee species: *Bombus terrestris*, *B. lucorum*, *B. pratorum*, and *B. pascuorum*. In a laboratory experiment testing the impacts of field relevant doses (1.87–5.32 ppb) of thimethoxam, they found that 2 weeks of exposure to the higher concentration of thiamethoxam caused a reduction in feeding in two out of four species, suggesting species-specific anti-feedant, repellency, or toxicity effects. The higher level of thiamethoxam exposure resulted in a reduction in the average length of terminal oocytes in queens of all four species. Further, the authors highlight that the discovery of species-specific effects on feeding has significant implications for current practices and policy for pesticide risk assessment and use.

Stingless bees (Apidae: Meliponini) are pan-tropical eusocial bees that are important pollinators for wild plants and crops (Barbosa et al. 2015). Little research on exposure and toxicology has been done for this diverse and abundant clade that is under pressure of habitat loss and intensification of agriculture. Lima et al. (2016) provide an overview of general agrochemical stressors on stingless bees.

Of the available studies involving neonicotinoids or fipronil, several indicate that the species studied are more sensitive to certain pesticides than *A. mellifera* and that results and testing procedures cannot be generalized. Topical LD50 (2.41 ng/bee 24 h, 1.29 ng/bee 48 h) and oral LC50 (2.01 ng/μL 24 h, 0.81 ng/μL 48 h) values for *Melipona scutellaris* for imidacloprid were lower than those of *A. mellifera* (Costa et al. 2015). Lourenco et al. (2012) found that for fipronil topical LD50 (0.6 ng/bee 48 h) and oral LC50 (0.011 ng/μL 48 h) were also lower than that of the honeybee. Rosa et al. (2016) found decreased larval survival feeding field-realistic doses (0.004 to 4.375 ng/larva) of thiamethoxam to *Scaptotrigona depilis* larvae in vitro. Low doses of fipronil (0.27 ng/bee topical, 0.24 ng/bee oral) affected brain morphology by apoptosis or necrosis of mushroom bodies of *Scaptotrigona postica* (Jacob et al. 2015), comparable to its effect on mushroom bodies of *A. mellifera* (Roat et al. 2014). Tomé et al. (2012) also found effects of imidacloprid on mushroom bodies and behavior in *Melipona quadrafasciata* and showed that imidacloprid impaired respiration and flight activity in this species. Valdovinos-Núñez et al. (2009) compared the toxicity of different pesticides for three stingless bee species (*Melipona beechei*, *Trigona nigra*, *Nannotrigona perilampoides*) and found neonicotinoids (imidacloprid, thiamethoxam, and thiacloprid) to be more toxic than permethrin and diazinon.

#### Synergistic effects of additional pesticides with neonicotinoids

Sgolastra et al. (2017) investigated the interaction between clothianidin and the ergosterol biosynthesis inhibiting (EBI) fungicide propiconazole in three bee species, *A. mellifera*, *B. terrestris*, and *O. bicornis*. Each species was administered a LD10 dose of clothianidin (0.86, 1.87, and 0.66 ng/bee, respectively, a non-lethal dose of propiconazole (7 μg/bee) and a combination of the two treatments. Bees were then observed for a 96-h period and mortality quantified. Some synergistic effects were recorded. In *A. mellifera*, mortality was significantly higher for the combined dose in the first two time periods (4 and 24 h). Mortality in *B. terrestris* for the combined dose was only significantly higher in the first time period, after 4 h. However, in *O. bicornis*, exposure to the combination of clothianidin and propiconazole resulted in significantly higher mortality at all time points.

Spurgeon et al. (2016) conducted similar experiments to Sgolastra et al., investigating the effect of a combination of clothianidin and propiconazole on *A. mellifera*, *B. terrestris*, and *O. bicornis*. In order to calculate an LD50, clothianidin concentrations were varied and propiconazole concentrations were held at zero, a low dose and a high dose. The low dose was taken from the EFSA Panel on Plant Protection Products (EFSA 2012) reported environmental concentrations, and the high dose was 10 times the low dose to represent a plausible worst-case scenario. Mortality was quantified over 48, 96, and 240 h. For *A. mellifera*, clothianidin LD50s with and without propiconazole were always within a factor of 2, with no clear negative trend at higher propiconazole concentrations. For *B. terrestris*, clothianidin LD50s with propiconazole were between 1.5- to 2-fold lower. For *O. bicornis*, clothianidin LD50s with propiconazole was up to 2-fold lower with a negative trend as propiconazole concentrations increased. Spurgeon et al. concluded that the clothianidin and propiconazole combination had no to slight synergy for *A. mellifera* and slight to moderate synergy for *B. terrestris* and *O. bicornis*.

In an additional trial, Thompson et al. (2014) demonstrated that the dose of fungicide applied is a key factor determining neonicotinoid toxicity using propiconazole and thiamethoxam mixtures. The authors argue that their low rates of significant synergies between neonicotinoids and fungicides was because of their lower, more field-realistic fungicide doses of 161–447 ng/bee compared to 10,000 ng/bee used by Iwasa et al. (2004), an early study demonstrating this interaction. The values of 161–447 ng/bee were calculated as realistic worst-case exposures based on approved application rates for UK crops. In a study of pesticide residues in pollen collected by *B. terrestris* in the UK, David et al. (2016) found concentrations of DMI fungicides up to 84 ppb, while Sanchez-Bayo and Goka (2014) report residues of propiconazole in honeybee-collected pollen up to 361 ppb. At the latter concentration a bee would need to consume approximately 28 g of pollen to receive the dose used in the Iwasa et al. (2004) study, which is not realistic. However, data are lacking demonstrating true field-realistic exposure rates to fungicides for free flying bees.

Overall, these studies support the position that neonicotinoids can act synergistically with fungicides, increasing their lethality to bees. However, the dose rate of both neonicotinoids and fungicides, time of exposure, neonicotinoid and fungicide chemical class, and length of time after exposure are all important explanatory factors affecting this relationship. The concentration of fungicide used in laboratory studies appears to be the most important factor determining synergistic lethality. Fungicides are regularly sprayed during the period when flowering crops are in bloom under the assumption that these compounds are safe for bees. Further work is needed in this area to establish realistic levels of chronic exposure to fungicides for free flying bees in order to assess the likely impact of neonicotinoid/fungicide synergies on bee populations.

Studies to date have only examined pairwise interactions between pesticides. It is clear that bees and other non-target organisms inhabiting farmland are routinely exposed to far more complex cocktails of pesticides than any experimental protocol has yet attempted to examine (e.g., David et al. 2016; Giorio et al. 2017 this special issue). A major challenge for scientists and regulators is to attempt to understand how chronic exposure to complex mixtures of neonicotinoids, fipronil, and other chemicals affects wildlife, this with or without other natural stressors (infectious agents, parasitism) and adverse abiotic conditions.

#### Population-level effects of neonicotinoids on wild bees

Nothing was known about the population-level effects of neonicotinoids on wild bees in 2014. As a managed domesticated species, population trend data are available for honeybees, but not for wild bees. One study has attempted to investigate the impact of neonicotinoids on wild bee population trends. Woodcock et al. (2016) used an incidence dataset of wild bee presence in 10 × 10 km grid squares across the UK. The dataset is comprised of bee sightings by amateur and professional entomologists and is probably the most complete national bee distribution database currently available. Sixty-two wild bee species were selected and their geographic distance and persistence over an 18-year period between 1994 and 2011 was calculated. Neonicotinoid seed-treated oilseed rape was first used in the UK in 2002, and so the authors calculated spatially and temporally explicit information describing the cover of oilseed rape and the area of this crop treated with neonicotinoids. The 62 species were split into two groups—species that foraged on oilseed rape (*n* = 34) and species that did not (*n* = 28). Species persistence across this time period was then compared with expected neonicotinoid exposure. Over the 18-year period, wild bee species persistence was significantly negatively correlated with neonicotinoid exposure for both the foraging and non-foraging group, with the effect size three times larger for the oilseed rape foraging group. Overall, the study suggests that bee species were more likely to disappear from areas with a high exposure to neonicotinoids as measured by the amounts applied as seed dressings to oilseed rape and that this trend was more pronounced for species known to forage on oilseed rape. While more work is needed, this is a major correlational study that suggests a link between levels of neonicotinoid exposure and bee community persistence at a national scale.

Rundlöf et al. (2015) conducted an extensive field trial of the effects of clothianidin-treated oilseed rape on wild bees. Sixteen oilseed rape fields separated by at least 4 km were selected across southern Sweden and were paired on the basis of similar landscape composition. In each pair, one of the fields was randomly selected to be sown with oilseed rape treated with 10 g clothianidin/kg of seed and the other field was sown without a neonicotinoid seed treatment. Twenty-seven cocoons of the solitary bee *O. bicornis* (15 male, 12 female) were placed out alongside each field a week before the oilseed rape began to flower, and six colonies of *B. terrestris* were placed alongside each field on the day the oilseed rape began to flower. The *O. bicornis* placed adjacent to treated oilseed rape showed no nesting behavior and did not initiate brood cell construction. *O. bicornis* adjacent to untreated fields showed nesting behavior in six of the eight fields studied. Bumblebees placed next to treated oilseed rape showed reduced colony growth and reproductive output. Bumblebee colonies were collected and frozen when new queens began to emerge, with this happening between the 7th of July and 5th of August depending on each colony. The number of queen and worker/male cocoons present was counted. At the point of freezing, colonies placed next to treated oilseed rape fields had significantly fewer queen and worker/male cocoons present.

Sterk et al. (2016) performed a similar field experiment to Rundlöf et al. Two areas of 65 km^2^ in northern Germany were selected in which the only flowering crops comprised of winter-sown oilseed rape. In one area, the oilseed rape was treated with the same seed coating used by Rundlöf et al. of 10 g clothianidin/kg seed. The other area was an untreated control. In each area, ten *B. terrestris* colonies were placed at each of six localities. Colonies were left adjacent to oilseed rape between April and June, covering its main flowering period. After this the colonies were moved to a nature reserve. No differences were found in colony weight growth, number of workers produced, or reproductive output as measured by the production of new queens.

It is interesting to note that the latter field studies, using the same neonicotinoid seed dressing, found markedly different results. The major difference is that while Rundlöf et al. used spring-sown oilseed rape, Sterk et al. used winter-sown oilseed rape. The length of time between sowing and peak flowering is much greater for winter-sown oilseed rape (mid-August to May) than for spring-sown oilseed rape (April/May to mid-June). As such, there is more time for neonicotinoids to degrade, and for them to leach into soil and water for winter-sown oilseed rape, reducing the amount of active ingredient available to be taken up by the crop. Indeed, the mean loads of clothianidin in the Rundlöf et al. study were 13.9 ppb in honeybee pollen, and 5.4 (bumblebee) and 10.3 (honeybee) ppb in nectars, whereas those in the German study were 0.50–0.97 ppb in honeybee pollen, 0.88 in bumblebee pollen, and 0.68–0.77 ppb in honeybee nectar (Rolke et al. 2016). Such a difference as revealed by exposure to the insecticide for honeybees (14–27 times less for pollen and 13–15 times less for nectar in the latter study) could explain the difference in reported colony growth and number of gynes and drones produced, since concentrations of clothianidin in the food of bees below 1 ppb are not supposed to produce any effect that were measured (Piiroinen et al. 2016). An additional difference is that in the Sterk et al. (2016) study, colonies were moved to a nature reserve consisting of forests, lakes, and heathland after the flowering period of oilseed rape ended. The quality of available foraging area at this nature reserve is likely to have been of both a higher quality and quantity than what was available in a conventional agricultural landscape and is not typical of the experience of a bumblebee colony located in such a landscape that will have to continue foraging there after crops such as oilseed rape cease flowering. In addition, a major problem with the experimental design of Sterk et al. is that only one treated and one control area were used, so there is no true site-level replication, as opposed to Rundlöf et al. who used eight treated and eight control fields. All these differences in experimental design highlight the difficulty of developing a single experimental design that may answer risk assessment questions for every potentially affected species. It also highlights the importance of evaluating the experimental design in terms of resulting data quality when considering the differences in results between Rundlöf et al. (2015) and Sterk et al. (2016).

Only one study is available that looked at the impact of neonicotinoids on the reproductive success of a solitary bee in controlled conditions. Sandrock et al. (2014a) established laboratory populations of *O. bicornis*, a solitary stem nesting bee. Bees were fed on sugar solution treated with 2.87 ppb thiamethoxam and 0.45 ppb clothianidin along with untreated pollen. There was no impact of neonicotinoids on adult female longevity or body weight. However, treated bees completed 22% fewer nests over the course of the experiment. Nests completed by treated bees contained 43.7% fewer total cells and relative offspring mortality was significantly higher, with mortality rates of 15 and 8.5% in the treated and untreated groups, respectively. Overall, chronic neonicotinoid exposure resulted in a significant reduction in offspring emergence per nest, with treated bees producing 47.7% fewer offspring. These results suggest that exposure to these low-level, field-realistic doses of neonicotinoids (< 3.5 ppb) did not increase adult mortality but did have sublethal impacts on their ability to successfully build nests and provision offspring.

#### Colony-level impact on bumblebees

Laycock et al. (2014) fed microcolonies of four *B. terrestris* workers thiamethoxam-treated sugar solution at a range of concentrations up to 98 ppb. Pollen was not treated with thiamethoxam. Sugar solution consumption was significantly reduced at the 39 and 98 ppb treatments. Worker mortality was only increased at the highest dose of 98 ppb. Worker oviposition failure was only significantly higher at the 39 and 98 ppb treatments, with no significant differences seen between the lower concentration treatments between 0 and 16 ppb.

Scholer and Krischik (2014) exposed greenhouse queenright colonies of *Bombus impatiens* to imidacloprid- and clothianidin-treated sugar syrup at concentrations of 0, 10, 20, 50, and 100 ppb for 11 weeks. Queen mortality was significantly increased at 6 weeks for the 50 and 100 ppb treatments and at 11 weeks for the 20 ppb treatment for both clothianidin and imidacloprid. Surprisingly, no significant impact was found on numbers of workers or new queens produced, though this was in part because very low numbers of new queens were produced across all treatments (average of four per colony). Colonies in treatments above 10 ppb imidacloprid and 20 ppb of clothianidin gained significantly less weight over the course of the study.

Cutler and Scott-Dupree (2014) placed *B. impatiens* colonies adjacent to maize fields during pollen shed in Ontario, Canada. Four neonicotinoid-treated conventional and four untreated organic fields were used. Colonies were placed adjacent to each field on the first day of major pollen shed. Colonies were left for 5–6 days and then transported to an area of semi-natural habitat for 30–35 days, after which they were frozen. Colonies placed next to treated maize produced significantly fewer workers than those placed next to organic farms. All other metrics (colony weight, honey and pollen pots, brood cells, worker weight, male and queen numbers and weights) were not significantly different. However, bumblebees collected less than 1% of their pollen from maize and neonicotinoid residues in collected pollen were very low (mean of 0.4 ppb) for bees foraging adjacent to treated fields and always below the LOD (0.1 ppb) for bees adjacent to organic fields. Given that it is well-known that bumblebees collect very low volumes of maize pollen, the relevance of this study is unclear.

FERA (2013) also conducted a field trial with *B. terrestris* colonies placed out adjacent to oilseed rape treated with either clothianidin, imidacloprid or an untreated control. Colonies were allowed to forage freely for 6–7 weeks while the oilseed rape flowered and then were moved to a non-agricultural area to continue developing. The initial aim was to measure colony growth and development across these three treatments and compare this with neonicotinoid concentrations collected from food stores within the nests, but the study was criticized for a number of methodological problems such as variable placement date and initial colony size, lack of site-level replication, and contamination of control colonies with neonicotinoid residues during the experiment. The study was ultimately not published in a peer reviewed journal but it came to the conclusion that there was no clear relationship between bumblebee colony success and neonicotinoid concentrations. Goulson (2015) reanalyzed the FERA data using linear models and retaining two colonies excluded in the original study as outliers, but which do not meet the statistical definition of this term. This reanalysis showed that the concentration of clothianidin in nectar (range 0 to 0.28 ppb) and the concentration of thiamethoxam in pollen (range 0 to 1.6 ppb) significantly and negatively predicted both colony weight gain and production of new queens. Very similar findings emerged from the recent large field trial of Woodcock et al. (2017) who exposed *B. terrestris* colonies to oilseed rape fields treated with either clothianidin, thiamethoxam or controls at field sites in the UK, Germany, and Hungary. Total neonicotinoid levels in the range 0 to 8 ppb in colony food stores negatively predicted colony reproductive output.

Most research on neonicotinoids and bees has focused on the three compounds subject to the EU moratorium. Thiacloprid is considered to be less dangerous to bees, since it has a much higher acute LD50. As a result, it is sometimes sprayed on crops or trees at or near flowering, potentially exposing bees to much higher doses than they would obtain from neonicotinoids applied as seed dressings. Ellis et al. (2017) placed *B. terrestris* colonies adjacent to raspberry crops that had been sprayed with thiacloprid following normal farming practice, and compared these to control nests placed next to unsprayed raspberry crops. Exposed colonies were more likely to die, grew more slowly, and produced 46% fewer reproductive than control colonies. This study strongly argues that thiacloprid should not be regarded as safe to bees.

Studies produced since 2014 have advanced our knowledge in several key areas. Laboratory studies have continued to demonstrate negative effects of neonicotinoids on bumblebee reproductive output at generally high concentrations, with the lowest sublethal effects on reproductive output detected at 10 ppb. Field studies using bumblebees demonstrate that exposure to neonicotinoid-treated flowering crops can have significant impacts on colony growth and reproductive output depending on the levels exposed to, with crop flowering date relative to sowing and availability of uncontaminated forage plants likely to explain variation in the detected residues among the available studies. Our understanding of the impact on solitary bees is much improved with the findings of Sandrock et al. (2014a) suggesting substantial impacts on solitary bee reproductive output at field-realistic concentrations of 3.5 ppb. Field studies demonstrating this under real-world conditions are limited with the work of Rundlöf et al. (2015) and Woodcock et al. (2017), demonstrating no nest-building activity at the neonicotinoid treatment sites.

Feltham et al. (2014) exposed *B. terrestris* colonies to sugar solution treated with 0.7 ppb and pollen treated with 6 ppb of imidacloprid for 2 weeks. Colonies were then placed out in an urban area in Scotland. The foraging workers from each nest were then monitored for a further 4 weeks. There was no difference in the length of time spent collecting nectar or the volume of nectar collected between workers from treated and control colonies. However, treated workers collected significantly less pollen, bringing back 31% less pollen per time unit to their colonies. Treated workers also collected pollen less frequently, with 41% of foraging bouts collecting pollen versus 65% for control workers; a decline of 24%.

Gill and Raine (2014) performed an experiment where *B. terrestris* colonies were exposed to sugar solution treated with 10 ppb of imidacloprid while also having access to forage freely outside. Colonies and individual worker bumblebees were studied over a 4-week period. In common with their previous findings (Gill et al. 2012), imidacloprid-treated workers initiated significantly more foraging trips across all 4 weeks of the experiment. The authors note that this is likely driven by an acute individual-level response in the first weeks (neonicotinoids acting as a neural partial agonist, increasing desire to forage) and by a chronic colony-level response in the latter part of the experiment, with treated colonies allocating a higher proportion of workers to pollen collection. Pollen foraging efficiency of treated workers decreased as the experiment progressed with the smallest collected pollen loads recorded in week four, suggesting a chronic effect of imidacloprid on pollen foraging ability. It is not clear whether this is as a result of individual performance deteriorating or new emerging workers having been exposed for a greater period of time.

Stanley et al. (2015a) exposed *B. terrestris* colonies to 2.4 or 10 ppb thiamethoxam-treated sugar solution for 13 days. Colonies were then moved to pollinator exclusion cages where they were allowed to forage freely on two varieties of apple blossom. Bees from colonies exposed to 10 ppb spent longer foraging, visited fewer flowers and brought back pollen on a lower proportion of foraging trips compared to bees from control colonies. Stanley and Raine (2016) also exposed *B. terrestris* colonies to 10 ppb thiamethoxam sugar solution for a 9- to 10-day period. At this point, colonies were moved to a flight arena provisioned with two common bird’s-foot trefoil (*Lotus corniculatus*) and one white clover (*Trifolium repens*) plants. Worker bees were individually released and their interaction with the flowers was recorded. Significantly more treated workers displayed pollen foraging behavior compared to control workers. However, control workers learned to handle flowers efficiently after fewer learning visits.

Arce et al. (2016) placed *B. terrestris* nests out in an area of parkland for a 5-week period while also supplying them with sugar solution treated with 5 ppb of clothianidin. The volume of sugar solution provided was estimated to be half that which colonies typically consume over the course of the experiment. No pollen was provided, so workers had to forage for this and to make up the shortfall in nectar resources. In contrast to the previous papers, only subtle changes to patterns of foraging activity and pollen collection were detected. There was no clear difference in colony weight gain between treatments or number of brood individuals. However, by the end of the experiment, treated colonies contained fewer workers, drones, and gynes when compared with control colonies.

Switzer and Combes (2016) studied the impact of acute imidacloprid ingestion on sonicating behavior of *B. impatiens*. Sonicating is a behavior whereby a bumblebee lands on a flower and vibrates loudly to shake pollen loose from anthers. Bumblebee workers were fed a dose of 0, 0.0515, 0.515, or 5.15 ng of imidacloprid in 10 μL of sugar solution. These are equivalent to concentrations of 0, 5.15, 51.5, and 515 μg/L (~ppb), with the highest volume consumed equivalent to 139% of the honeybee acute LD50, a moderate proxy for bumblebees, as bumblebees are generally less sensitive than honeybees. Bees were then allowed to forage from tomato (*Solanum lysopersicum*) plants and sonicating behavior was observed. At the lowest dose of 5.15 μg/L of imidacloprid, no impact was found on wingbeat frequency, sonication frequency or sonication length. No analysis could be made for higher doses, as bees in these treatments rarely resumed foraging behavior after ingesting imidacloprid. Given the neonicotinoid concentrations used in this study and sample size problems, it is difficult to draw many conclusions other than that levels of exposure above 50 μg/L impair bumblebee pollen foraging behavior.

Overall, these studies suggest that exposure to neonicotinoids in nectar at concentrations between 0.7–10 ppb can have sublethal effects on the ability of bumblebees to collect pollen at both the individual and colony level. This shortfall in pollen and subsequent resource stress is a plausible mechanism to explain diminished colony growth and production of sexuals in the absence of increased direct worker mortality. Given that concentrations as high as 10 ppb are at, but within, the upper limit of what bumblebees are likely to experience in the field, it is likely that wild bumblebees exposed to neonicotinoids in contemporary agricultural environments suffer from a reduced ability to collect pollen, with a subsequent impact on their reproductive output.

### Effects of neonicotinoids and fipronil on other invertebrates

#### Effects on target pests

Fipronil induced *Drosophila* S2 cell apoptosis in vitro experiments (Zhang et al. 2015). This side effect occurs through caspase-dependent mitochondrial pathways and appears to coincide with a decrease in the mitochondrial membrane potential and an increase in reactive oxygen species. Other authors have shown significant increases in tumor frequencies on wing cells of *Drosophila melanogaster*, suggesting that this insecticide is mutagenic and carcinogenic in somatic cells of this fruit fly (de Morais et al. 2016a).

Wild strains of *Drosophila melanogaster* are rather resistant to imidacloprid with acute LD50s > 1304 μM (> 333.8 ppm) for both females and males (Charpentier et al. 2014). However, the same study has shown lethal effects of imidacloprid on chronically exposed *D. melanogaster* during 8 days: 27% of females died at 3.91 nM and 28% of males at 39.1 nM*.* The latter concentrations were several orders of magnitude below chronic LC50s of 18 and 45 μM for females and males, respectively. Moreover significant sublethal effects have been demonstrated on mating and fecundity at very low exposure concentrations (mating: both genders exposed at 0.391 nM; fecundity: females exposed at 3.91 nM), i.e., in the 0.1–1 ppb range of concentration.

Stimulated reproduction of the green peach aphid (*Myzus persicae*) by exposure to sublethal doses of imidacloprid had been reported previously (Yu et al. 2010). This hermetic effect undermines the effectiveness of the insecticide in controlling the target pest, and it seems to be accompanied by a complex pattern of up- and downregulation of genes during exposure. A recent study suggests that such an effect is passed on to the second generation, although there is some adaptability to low doses of the insecticide (Ayyanath et al. 2014). In another study, the soybean aphid (*Aphis glycines*) showed significantly higher reproduction rate when treated at sublethal doses of imidacloprid (0.05 mg/L) than in non-treated controls (Qu et al. 2015). However, other sublethal doses (0.1 and 0.2 mg/L) caused slower juvenile development, shorter reproductive periods, and reduced adult lifespan and fecundity, indicating that the threshold for hormetic responses is rather low. Stimulatory reproductive effects have also been observed with exposure of males of the Neotropical brown stink (*Euschistus heros*) to imidacloprid, but not with exposure of the females (Haddi et al. 2016).

A commercial mixture of a pyrethroid (b-cyfluthrin) and a neonicotinoid (imidacloprid) produced behavioral sublethal effects on bed bugs (*Cimex lectularius*) such as reduced locomotion, reduced feeding, and difficulties in host-finding that resulted in good control of the bugs by preventing their dispersal (Crawley et al. 2016). However, the bugs have already developed resistance to at least four neonicotinoids, acetamiprid, dinotefuran, imidacloprid, and thiamethoxam in several North American cities (Romero and Anderson 2016).

Efficacy of three neonicotinoids (acetamiprid, imidacloprid, and thiamethoxam) on controlling sand termites (*Psammotermes hypostoma*; Isoptera) has been demonstrated by Ahmed et al. (2015), the effect of all three lasting up to 60 days. Dembilio et al. (2015) also studied the lasting effect of imidacloprid applied by crown spray or stipe injection on palms to control the red palm weevil (*Rhynchophorus ferrugineus*; Coleoptera). Complete control (100%) was achieved after 45 days of stipe injections at 4–10 mL, corresponding to 2 g a.i./plant. Spray application used larger volumes that stipe injections, were less efficient and resulted in larger losses of the insecticide by washoff from the palm fronds into the surrounding environment. In Brazil, plantations of eucalypts were treated by immersing the seedlings in a solution containing fipronil (0.4%) to prevent attack by termites, as it has been shown to be protective for 56 days (dos Santos et al. 2016). A comparison of termites’ diversity between treated and untreated plots did not show significant differences, although treated plots tended to have fewer species (Silva et al. 2016). The authors indicated that “any effect [of fipronil] is masked by the effects of the plantation itself”, since both treated and untreated plantations of this tree had significantly less termite diversity than native savanna forests or regeneration forests.

Baits laced with fipronil are being used to control the expansion of the invasive Argentine ant (*Linepithema humile*) in Japan. Although the main super colonies of this species appear to be very susceptible to this insecticide, the treatment was also damaging for all other local ant populations. Fipronil bait treatments, therefore could lead to significant impacts on the local arthropod biodiversity (Hayasaka et al. 2015). Hydrogels containing 750 ppb thiamethoxam killed 50% of the forager ants in 3 days, while baiting with 1500 and 750 ppb provided 100% mortality of workers and queens within 8 days in the laboratory trials. These concentrations were lower than the ones required to control ants in highly infected areas as reported by Rust et al. (2015). At sublethal levels, imidacloprid may have different effects in red imported fire ants (*Solenopis invicta*) depending on the concentrations used; for example, concentrations of this insecticide in sugar water at 0.01 ng/L are attractive to the ants and increase their digging activity, whereas concentrations higher than 0.25 ng/L suppress their water consumption, digging and foraging behaviors (Wang et al. 2015e). At the latter concentration, newly mated queens reduced their brood tending ability, while the time to larval emergence was delayed significantly and no pupae or adult workers were produced (Wang et al. 2015d).

#### Effects on butterflies

Mulé et al. (2017) presented a systematic review of the effects of chemical insecticides on four common butterfly families: *Lycaenidae*, *Nymphalidae*, *Hesperiidae*, and *Papilionidae*. Only one study in their sample (Krischik et al., 2015) looked at the effects of a neonicotinoid (imidacloprid) on butterflies (*Danaus plexippus* and *Vanessa cardui*) illustrating a huge data gap. The systematic review concludes that the use of all the insecticides studied (dichlorvos, imidacloprid, malathion, naled, permethrin, and resmethrin) cause negative effects on the most common butterfly families, such as reduced survival rate, feeding interruption, and alteration of oviposition behavior.

#### Effects on natural enemies of pests

Compared to the research on pollinators, few studies on the toxic effects and population impacts of neonicotinoids and fipronil on other arthropods have been published in the past 2 years. Research in this case has been limited to beneficial insects used in biological control or integrated management programs (IPM), building upon the already known negative effects of these chemicals (Pisa et al. 2015). Recent studies have centered on the newly developed compounds (Giorio et al. 2017, this special issue), while the previous literature focused mainly on imidacloprid.

### Predators

The efficacy of neonicotinoids and fipronil for pest control and the negative effects they inflict on beneficial predators are directly correlated. Both effects depend on the toxicity to pest and to their predators and on the residue level of exposure in plants (which have been treated or not). It has been shown that uptake of several neonicotinoids after seed treatment in cotton crops differed according to their water solubility, with nitenpyram, dinotefuran, and thiamethoxam showing the highest residues in plant tissues and lowest in the soil (Zhang et al. 2016b). Consequently, these three compounds were more effective against the cotton aphid *Aphis gossypii* than the other four neonicotinoids. However, residues in soil of all seven neonicotinoids reduced the soil fauna significantly (*p* < 0.05), in particular the larvae of hoverflies (Diptera: Syrphidae). Foliar sprays of all compounds produced similar effects to their seed treatments but the impacts on soil larvae were not significant (Zhang et al. 2016b). The authors acknowledge the efficient aphid control by the three neonicotinoids above while warning of the long-term negative effects derived of suppressing beneficial insect’s larvae and also species that feed on extra floral nectar, such as some ladybugs and polyphagous parasitoids. Another study suggests that not only are there no significant negative impacts of seed-treated imidacloprid and clothianidin on the beneficial insects of winter wheat crops (i.e., ladybirds, hoverflies, or parasitoids) but both systemic treatments increased the density of spiders, despite residues in plants being present for 200 days (Zhang et al. 2016a). The latter field study was conducted throughout the winter season in northern China, with planting in October and harvest in June, when the soil larvae were dormant or going through diapause—hence the lack of negative effects, contrasting with those observed in summer crops and the high toxicity of neonicotinoids to coccinelid larvae (Lucas et al. 2004).

The variability of impacts on natural enemies of crop pests depends on the intrinsic toxicity of the active ingredients and co-formulants, and the rate of application to the crop. Thus, cotton plants grown from seeds treated with thiamethoxam at the recommended doses (3 g/kg) reduced the populations of natural enemies of the cotton leafhopper by about 35%, in particular those of *Chrysoperla* sp., *Orius* sp., and spiders, whereas cotton plants grown from seeds treated with imidacloprid (5 g/kg) did not lead to significant reductions (i.e., < 10%) of the same species (Saeed et al. 2016). Trials in South Dakota demonstrated the effectiveness of seed treatments of thiamethoxam and imidacloprid, whether alone or in combination with foliar sprays of beta-cyflutrin, for controlling aphids. However, thrips (Thysanoptera) increased in number in one of the locations regardless of the treatment used, and this effect was significantly correlated with their major predators, as several taxa of natural enemies declined (Regan et al. 2017).

Polyphagous ladybird beetles like *Coleomegilla maculata* and *Hippodamia convergens* feed occasionally on the nectar of sunflowers. Three routes of exposure of thiamethoxam for the ladybird *Serangium japonicum* that controls the whitefly *Bemisia tabaci* were tested. Predation of *S. japonicum* was most reduced under systemic exposure and least by contact with residues on the surface, following the same pattern as the lethal toxicity (Yao et al. 2015). In a similar way, oral exposure of the ladybird *Eriopis connexa* to acetamiprid at maximum recommended rates (200 mg/L) in water resulted in 90% mortality of adults after 15 days, while the survival of pupae treated in Petri dishes at half that rate (100 mg/L) was reduced only up to 15%. However, 83% of the emerging adults had a number of malformations (Fogel et al. 2016). Residues of imidacloprid and thiamethoxam on filter paper appear to repel the predatory beetles *Cycloneda sanguinea* and *Chauliognathus flavipes* as well as the predatory bug *Orius insidiosus* (Fernandes et al. 2016), but tomato leaves and plants treated with foliar sprays of imidacloprid at 100 ppm had residual activity and caused 62% mortality of the mirid bug *Macrolophus basicornis* a month after treatment (Wanumen et al. 2016a). The life span of *Coccinella septempunctata* adults exposed to sublethal doses of imidacloprid sprayed on leaves (4.8 ppm) was reduced by 24–28%, while their fecundity was reduced by 53–56% and the oviposition period was shortened significantly. Moreover, the fecundity of the F1 generation was also reduced considerably (Xiao et al. 2016). Foliar sprays of imidacloprid on okra crops in India at recommend label rates (21–24.5 g/ha) reduced the populations of spiders and ladybeetles significantly in the first 2 weeks after spraying, but the authors concluded that the insecticide was safe to natural enemies because they recovered after a while (Karthik et al. 2015).

Unfortunately, the rates of application of neonicotinoids in greenhouses, nurseries, and trees in urban landscapes are much higher than the rates applied to field crops. Thus, imidacloprid applied at 300 mg/L to pots containing the Mexican milkweed (*Asclepias curassavica*) resulted in very high concentrations of 6 ppm in the flowers after one application and 21 ppm after a second one done 7 months later. Consequently, the concentrations of imidacloprid in pollen of the nursery flowers were 793 to 1368 times higher than the typical residues (7.6 ppb) obtained from seed-treated canola plants. Such residue levels caused significant mortality in three lady beetle species after 12 days, *Coleomegilla maculata* (50–65%)*, Harmonia oxyridis* (25–50%), and *Hippodamia convergens* (30–50%), but less in *Coccinella septempunctata* (~ 10–15%). Caterpillars of *Danaus plexippus* fed on the same plants experienced > 90% mortality after 1 week and were wiped out after 3 weeks. *Vanessa cardui* butterflies fed on flowers of globe thistle (*Echinops ritro*) treated at the same rate experienced mortalities of over 30% after 1 week compared to untreated controls (Krischik et al. 2015).

In field experiments, sunflowers grown from seeds treated with thiamethoxam at the recommended dose (0.5 mg/kg seeds) did not cause significant mortality of the predatory bug *Orius insidiosus*, but reduced its egg viability and female fertility resulting in a 40% reduction in nymph survival (Gontijo et al. 2015). The same treatment, however, caused 48% mortality of the predator *Chrysoperla carnea* after 8 days exposure (Gontijo et al. 2014). Thiamethoxam applied as seed treatment also delayed emergence of *Coleomegilla maculata* by prolonging the pupal period, whereas it reduced egg viability and skewed the sex ratio of *Hippodamia convergens* in favor of females (Moscardini et al. 2015).

Secondary poisoning has been shown with second instars of the ladybug *Coleomegilla maculata*, which have slower walking and predatory skills when feeding on aphids (*Rhophalosiphum padi*) grown on wheat plants seed-treated with thiamethoxam. Interestingly, only residues of its metabolite clothianidin were found in the aphids (Bredeson et al. 2015). The omnivorous mirid predator *Nesidiocoris tenuis* experienced a mortality of 36% when feeding on eggs of *Ephestia kuehniella* (Lepidoptera, Pyralidae) that had been treated with sulfoxaflor at the highest recommended rates (60 mg/L) for controlling this pest. In addition, fecundity and longevity of the predatory bug were reduced significantly, indicating that this member of the fourth generation of neonicotinoids has undesirable sublethal effects on natural enemies (Wanumen et al. 2016b).

An update on the acute toxicity of seven neonicotinoids and fipronil to predatory arthropods is shown in Table 1. As most of the authors cited above have indicated, the sublethal effects of thiamethoxam on reproduction and predation ability of natural enemies and the residual activity of imidacloprid do not warrant the use of these insecticides in IPM programs.

**Table 1:** Lethal median concentrations (LC_50_, mg/L) of systemic insecticides to predatory arthropods of crop pests

Scientific name	Taxon	Acetamiprid	Clothianidin	Dinotefuran	Imidacloprid	Thiacloprid	Thiamethoxam	Fipronil	References
*Neoseiulus fallacis*	Acari: Phytoseiidae		57						Lefebvre et al. (2012)
*Phytoseiulus macropilis*	Acari: Phytoseiidae				3561				Mizell and Sconyers (1992)
*Gnathonarium exsiccatum*	Arachnida: Linyphiidae				801				Tanaka et al. (2000)
*Ummeliata insecticeps*	Arachnida: Linyphiidae				995				Tanaka et al. (2000)
*Pardosa pseudoannulata*	Arachnida: Lycosidae				440				Tanaka et al. (2000)
*Pardosa pseudoannulata*	Arachnida: Lycosidae				40.4				Chen et al. (2012)
*Tetragnatha maxillosa*	Arachnida: Tetragnathidae				136				Tanaka et al. (2000)
*Chauliognatus flavipes*	Coleoptera: Cantharidae				80*		470*		(Fernandes et al. 2016)
*Adalia bipunctata*	Coleoptera: Coccinellidae				218.9		232		Amirzade et al. (2014)
*Adalia bipunctata*	Coleoptera: Coccinellidae				74				Jalali et al. (2009)
*Cheilomenes quadriplagiata*	Coleoptera: Coccinellidae							307	Wu et al. (2007)
*Coccinella septempunctata*	Coleoptera: Coccinellidae				35.8				Xue and Li (2002)
*Coccinella septempunctata*	Coleoptera: Coccinellidae				726				Bozsik (2006)
*Coccinella undecimpunctata ssp. aegyptica*	Coleoptera: Coccinellidae	93.5			34.2				Ahmad et al. (2011)
*Coccinella undecimpunctata ssp. aegyptica*	Coleoptera: Coccinellidae	263.4			447.8		296.6		Amirzade et al. (2014)
*Cryptolaemus montrouzieri*	Coleoptera: Coccinellidae				20.6				Khani et al. (2012)
*Cycloneda sanguinea*	Coleoptera: Coccinellidae				760*		420*		Fernandes et al. (2016)
*Harmonia axyridis*	Coleoptera: Coccinellidae	< 4–16.7			30.3–364		153.3		Youn et al. (2003)
*Hippodamia convergens*	Coleoptera: Coccinellidae				161.4			164.3	Kaakeh et al. (1996)
*Hippodamia variegata*	Coleoptera: Coccinellidae						788.5		Rahmani and Bandani (2013)
*Olla v-nigrum*	Coleoptera: Coccinellidae				3.07				Mizell and Sconyers (1992)
*Propylaea japonica*	Coleoptera: Coccinellidae							629	Wu et al. (2007)
*Propylaea sp.*	Coleoptera: Coccinellidae				12.4				Xue and Li (2002)
*Serangium japonicum*	Coleoptera: Coccinellidae						2.43		Yao et al. (2015)
*Stethorus japonicus*	Coleoptera: Coccinellidae				0.6				Mori and Gotoh (2001)
*Orius insidiosus*	Hemiptera: Anthocoridae				2.78		1.67		Prabhaker et al. (2011)
*Orius insidiosus*	Hemiptera: Anthocoridae				80*		380*		Fernandes et al. (2016)
*Orius laevigatus*	Hemiptera: Anthocoridae				0.04–0.3				Delbeke et al. (1997)
*Geocoris punctipes*	Hemiptera: Lygaedae				5180		2170		Prabhaker et al. (2011)
*Cyrtorhinus lividipennis*	Hemiptera: Miridae				0.36				Tanaka et al. (2000)
*Cyrtorhinus lividipennis*	Hemiptera: Miridae		0.043		0.94				Preetha et al. (2010)
*Deraeocoris nebulosus*	Hemiptera: Miridae				0.0163				Mizell and Sconyers (1992)
*Hyaliodes vitripennis*	Hemiptera: Miridae	0.7			1.1	0.3	0.5		Bostanian et al. (2005); Bostanian et al. (2001)
*Podisus maculiventris*	Hemiptera: Pentatomidae				4.7				De Cock et al. (1996)
*Podisus maculiventris*	Hemiptera: Pentatomidae				5				Cutler et al. (2006)
*Podisus nigrispinus*	Hemiptera: Pentatomidae				0.285		0.055		Torres and Ruberson (2004)
*Chrysoperla rufilabris*	Neuroptera: Chrysopidae				121.7				Mizell and Sconyers (1992)
*Scolothrips takahashii*	Thysanoptera: Thripidae				1.81				Mori and Gotoh (2001)

* μg/cm^2^

### Parasitoids

A “meta-analysis of nearly 1,000 observations from North American and European field studies revealed that seed-applied neonicotinoids reduced the abundance of arthropod natural enemies similarly to broadcast applications of pyrethroid insecticides” (Douglas and Tooker 2016). The study also indicates that seed-applied neonicotinoids are less toxic than pyrethroids to spiders and mites, so they might contribute to biological control in some particular agricultural systems.

Imidacloprid, dinotefuran, and thiamethoxam were more toxic to the filth fly parasitoid *Spalangia endius* (Hymenoptera: Pteromalidae) than to the target pest (*Musca domestica)*, thus making it unsuitable for controlling the flies (Burgess and King 2015). Also *S. endius* was attracted to imidacloprid granular baits and experienced more grooming activity, but not to those containing dinotefuran or other insecticides. By contrast, another Pteromalidae parasitoid, *Urolepis rufipes*, did not show that behavior with either neonicotinoid tested (Burgess and King 2016). In semi-field tests designed to evaluate the toxicity of 19 new insecticides applied at label rates to cotton on the egg parasitoid *Trichogramma pretiosum*, fipronil (480 ppm) and dinotefuran (1040 ppm) showed almost 100% mortality in 24 h, whereas acetamiprid (429 ppm) caused 80% mortality (Khan et al. 2015). Similarly, imidacloprid applied at the label rate on citrus trees (40 ppm) is harmful to the encyrtid parasitoid *Ageniaspis citricola*, causing over 89% mortality in 24 h and having residual activity on the leaves up to 17 days (de Morais et al. 2016b). Adults of the parasitoid *Tamarixia triozae* (Eulophidae) experienced 28 to 58% mortality after contact with pepper leaves that had been sprayed with imidacloprid (range 3 to 260 ppm); the highest dosage also caused cumulative 100% mortality after 2 days, whereas the lower treatments reduced emergence by 26–63% in a dose-related manner (Martinez et al. 2015). However, residues of imidacloprid on sprayed tomato leaves (1155 ppm) caused only 38% mortality in 24 h to the parasitoid *Tamarixia triozae* (Eulophidae), although its residual activity after 11 days was still noticeable and still caused 25% mortality (Luna-Cruz et al. 2015).

Laboratory tests using glass residues (contact toxicity) have demonstrated the high toxicity of imidacloprid, dinotefuran, nitenpyram, and thiamethoxam to the egg parasitoid *Trichogramma ostrinae* (Table 2), and all neonicotinoids except nitenpyram appear to pose a high risk in IPM: the fecundity is reduced by more than 50% in the case of thiamethoxam and dinotefuran, while emergence is reduced 54% in the case of imidacloprid exposure (Li et al. 2015b). In the case of *Trichogramma chilonis* exposed to residues of commercial formulations of thiamethoxam and nitenpyram, mortalities of 98 and 96%, respectively, were observed in 24 h. This together with a reduction of parasitism in the larval stage: 20–37% for thiamethoxam and 14–45% for nitenpyram, and a reduced emergence of 12–33% for thiamethoxam and 21–29% for nitenpyram (Ko et al. 2015). Also, exposures by contact following the recommended paper disc tests of the International Organization for Biological Control (IOBC) showed the high acute toxicity of thiamethoxam, imidacloprid and acetamiprid when applied at 25, 30–40, and 60 ppm concentrations. Adult mortalities of the parasitoid *Tamarixia radiata* after 3 days were 100, 61–78, and 66% for the respective insecticides (Beloti et al. 2015). Therefore, these insecticides were classified in Class 4 (harmful) and not recommended for IPM programs (Veire et al. 2002).

**Table 2:** Lethal median concentrations (LC_50_, mg/L) of systemic pesticides to Hymenoptera parasitoids of crop pests

Scientific name	Family	Acetamiprid	Clothianidin	Dinotefuran	Imidacloprid	Nitenpyram	Thiacloprid	Thiamethoxam	Fipronil	References
*Aphelinus mali*	Aphelinidae				0.16					(Cohen et al. 1996)
*Aphytis melinus*	Aphelinidae	0.005			0.246			0.105		(Prabhaker et al. 2011; Prabhaker et al. 2007)
*Encarsia formosa*	Aphelinidae	12			0.98			0.397		Prabhaker et al. 2007 and 2011
*Encarsia inaron*	Aphelinidae				208.9					(Sohrabi et al. 2013)
*Eretmocerus eremicus*	Aphelinidae	108.3			1.93			1.01		Prabhaker et al. 2007 and 2011
*Eretmocerus mundus*	Aphelinidae				4.75					Sohrabi et al. 2013
*Apanteles subandinus*	Braconidae				530					(Symington and Horne 1998)
*Aphidius colemani*	Braconidae				0.327					(Charles-Tollerup 2013)
*Bracon hebetor*	Braconidae								0.082**	(Danfa et al. 1998)
*Cotesia chilonis*	Braconidae								0.0067	(Huang et al. 2011)
*Cotesia vestalis*	Braconidae								0.475	(Wu and Jiang 2004)
*Diaeretiella rapae*	Braconidae				5.1				0.4	(Wu et al. 2004)
*Opius flavus*	Braconidae								0.059	(Wu et al. 2007)
*Orgilus lepidus*	Braconidae				50					Symington and Horne 1998
*Psyttalia concolor*	Braconidae				~ 150				< 40	(Adán et al. 2011)
*Syngaster lepidus*	Braconidae				0.288					(Paine et al. 2011)
*Haplogonatopus sp.*	Dryinidae				0.12					(Tanaka et al. 2000)
*Avetianella longoi*	Encyrtidae				0.212					Paine et al. 2011
*Copidosoma koehleri*	Encyrtidae				48					Symington and Horne 1998
*Ooencyrtus nezarae*	Encyrtidae							50		(Alim and Lim 2014)
*Neochrysocharis okazakii*	Eulophidae		0.0231		0.0035					(Tran and Ueno 2012)
*Oomyzus sokolowskii*	Eulophidae	35.183								(Cordero et al. 2007)
*Diadegma insulare*	Ichneumonoidae				41.5					(Hill and Foster 2000)
*Diadegma insulare*	Ichneumonoidae	23.9								Cordero et al. 2007
*Diadromus collaris*	Ichneumonoidae								0.12	Wu et al. 2007
*Anagrus nilaparvatae*	Mymaridae				0.021			0.52	0.18	(Wang et al. 2008)
*Anaphes iole*	Mymaridae				0.053**			1.7		(Williams III L et al. 2003)
*Gonatocerus ashmeadi*	Mymaridae	0.134			2.63			1.44		Prabhaker et al. 2007 and 2011
*Trissolcus nigripedius*	Platygastridae							500		(Lim and Mahmoud 2008)
*Catolaccus grandis*	Pteromalidae								0.087	(Elzen et al. 1999)
*Pteromalus puparum*	Pteromalidae								0.11	Wu et al. 2007
*Spalangia endius*	Pteromalidae			52.2 *	17.92 *			41.94*		(Burgess and King 2015)
*Trichomalopsis sp.*	Pteromalidae								0.2	Wu et al. 2007
*Urolepis rufipes*	Pteromalidae			0.8 *	10.*					(Burgess and King 2016)
*Gryon japonicum*	Scelionidae							500		Alim and Lim 2014
*Haeckeliania sperata*	Trichogrammatidae				423					(Carrillo et al. 2009)
*Trichogramma cacoeciae*	Trichogrammatidae				1.25					(Saber 2011)
*Trichogramma chilonis*	Trichogrammatidae		0.0113		0.0027			0.0014		(Preetha et al. 2009)
*Trichogramma chilonis*	Trichogrammatidae								0.376	(Wang et al. 2012a)
*Trichogramma confusum*	Trichogrammatidae	93.2			754.2	0.84	176.5	0.24	0.86	(Wang et al. 2013)
*Trichogramma evanescens*	Trichogrammatidae	24.46			50.28	2.9	17.24	1.12		(Wang et al. 2014)
*Trichogramma japonicum*	Trichogrammatidae	25.39			95.48		75.26	0.4	0.92	(Zhao et al. 2012)
*Trichogramma nubilale*	Trichogrammatidae	19.2			312	4.37	56.73	1.86	0.29	(Wang et al. 2012c)
*Trichogramma nubilale*	Trichogrammatidae	0.609			2					(Chen et al. 2013)
*Trichogramma ostriniae*	Trichogrammatidae			0.12	2.94	1.58		0.14		(Li et al. 2015b)
*Trichogramma ostriniae*	Trichogrammatidae	43.02			503.6	4.93	376.3	2.48	0.14	(Wang et al. 2012b)
*Trichogramma pretiosum*	Trichogrammatidae							0.53 §		(Williams III L and Price 2004)

*ng/cm^2^

**kg/ha

^§^LC90

Furthermore, females of the chalcid wasp *Nasonia vitripennis* exposed to sublethal concentrations of imidacloprid in syrup (2–100 ppb) not only experienced 20–25% reduced fecundity but also altered the sex allocation of the offspring in favor of females while reducing the fitness when ovipositing with co-foundresses (Whitehorn et al. 2015). The reproductive impairment observed so far with various species of parasitoid wasps is evidence that links neonicotinoids to the decline of these beneficial and important species for pest control.

#### Effects on non-target soil organisms

Imidacloprid and thiacloprid are highly toxic to springtails (Collembola spp.), with LC50s of 0.44 and 9 mg/kg dry soil, respectively, for *Folsomia candida*. In multigenerational tests with this species, imidacloprid showed consistently high toxicity through three generations, whereas toxicity of thiacloprid was reduced in the second and third generation. The authors suggest that the higher persistence of imidacloprid in soil compared to that of thiacloprid could be the reason for this differential toxicity in time (van Gestel et al. 2017).

The only microcosm study available for terrestrial arthropods using imidacloprid was done by Uhl et al. (2015). The experimental setup consisted of a tritrophic system: strawberry plants, a ground cricket (*Nemobius sylvestris*) and a web spider (*Pisaura mirabilis*). Strawberry leaves were treated at two different rates that reflected a typical dosage within the crop (2.4 g/m^2^) and low exposure in field margins and forests (0.24 g/m^2^). The treatments were sublethal, as cricket mortalities were low and evenly distributed among treatments and controls. However, crickets showed significantly less mobility and feeding behaviors with the high treatment, while both treatments resulted in significantly lower weight and thorax growth. The high treatment also increased the predation of crickets by the spider, while spiders tended to move more under such circumstances. However, and surprisingly, the low treatment resulted in higher survival of the crickets than in the controls. Overall, herbivory was reduced and predation increased at sublethal concentrations of imidacloprid, suggesting possible impacts through trophic interactions.

Significant changes in invertebrate community composition were observed immediately after spray applications of fipronil for locust (*Chortoicetes terminifera*) control in Queensland, Australia, though the richness and abundance of species caught in pan and pitfall traps were not significantly affected. The changes in species composition for the flying insects (pan traps) persisted for up to 79 days after spraying operations, whereas those for the ground-dwelling invertebrates (pitfall traps) lasted up to 189 days. The authors of this field study explained that a long drought period that occurred during their 2-year monitoring may have influenced the slow recovery of the invertebrate populations (Walker et al. 2016). The composition of arthropod communities was not significantly affected over time in another study that used fipronil sprays for locust control in New South Wales, Australia (Maute et al. 2017a). However, springtails, mites, beetles, crickets, psocopterans, and dipterans experienced short-term decreases in abundance. The highest reductions were observed in two ant species, one of which did not recover for longer than a year. Because arthropod abundance and community assemblage changed over the 2-year study, both in control and treatment sites, changes in patterns of local rainfall over the study period were larger than changes in abundance due to pesticide treatment (Maute et al. 2017a). The same authors found no impacts on wood-eating termites activity, measured as consumption of wooden baits, or species composition but such termites are soil dwellers, are scarce in arid Australia and may not have been exposed to the sprays (Maute et al. 2016). Equally, litter decomposition carried out by soil microbial communities was not affected by the fipronil sprays (Maute et al. 2017b).

The few species of earthworms used in toxicology tests are more tolerant to neonicotinoids than other soil invertebrates. However only few species have been studied and *Eisenia fetida*, the species on which most studies were performed is an epigean (i.e., surface-dwelling) compost worm and is not a common species in forest or agricultural soil. The acute toxicities (LC50s in mg/kg soil) of five neonicotinoids to the earthworm *Eisenia fetida* after 14 days exposure were determined as 4.34 for nitenpyram, 3.05 for imidacloprid, 2.69 for acetamiprid, 2.68 for thiacloprid, and 0.93 for clothianidin (Wang et al. 2015b). The authors reported that exposures in the range 0.8–2.0 mg/kg also reduced the fecundity of this species between 39.5 and 84% depending on the compounds, while causing significant disruption of epidermal and midgut tissues. The median number of hatched cocoons (EC50) for earthworms exposed to imidacloprid was determined as 0.92 mg/kg soil, and its lowest observed effect concentrations (LOECs) for hatchability, AChE activity, growth, and DNA damage were 0.02, 0.1, 0.5, and 0.5 mg/kg soil, respectively (Wang et al. 2015c). Mixtures of imidacloprid and lambda-cyhalotrin appear to have antagonistic toxicity in these earthworms (Wang et al. 2015f). Other authors have demonstrated the low toxicity of the new compound guadipyr to *Eisenia fetida*, as concentrations of this insecticide in soil below 100 mg/kg in did not affect the growth or the reproduction output. Only increases in enzymatic activities of superoxidase dismutase and catalase were observed in the first few days of exposure, returning to normal levels afterwards (Wang et al. 2015a). The 48-h LC50 for acute toxicity of nitenpyram to the earthworm *Pheretima posthuma* was determined at 0.29 mg/kg soil (Hussain et al. 2017).

A study of the nematode communities in soils of corn fields treated with clothianidin, either as granules or in seed-coating, found significant differences in species richness and diversity compared to untreated control fields on two separate years. The main driver in community composition of nematodes was the year and month of sampling, but clothianidin treatments reduced the diversity of species significantly, with up to 5 species out of 36 being absent, even if total abundances between treatments and controls were statistically similar (Čerevková et al. 2017).

The enantiomers of fipronil appear to have different toxicity to the earthworm *Eisenia fetida*, with S-fipronil having a higher subchronic toxicity and bioaccumulation potential than R-fipronil (Qin et al. 2015). Weight reductions after 28 days exposure to concentrations in soil ranging 50–1000 mg/kg were 23–53% for the R-fipronil and 38–62% for the S-fipronil enantiomers. Residue accumulation in the earthworms reached a peak after 10 days exposure and then declined, following the dissipation pattern of the initial fipronil racemate in the soil. In tissues, fipronil, fipronil sulfone, and fipronil sulfide were detected, with bioaccumulation factors of 0.5–0.75 and a biological half-life in the range 1.5–2.1 days (Qin et al. 2015).

Studies on the effect of systemic pesticides on soil organisms are limited to a few species and the chosen species may not be the most ecologically meaningful. Therefore, the true impact of systemic pesticides on soil organisms and associated functions remains an important knowledge gap.

#### Effects on aquatic invertebrates

A comprehensive review of the acute and chronic toxicity of neonicotinoids to 49 species of aquatic insects and crustaceans, spanning 12 invertebrate orders, indicates that differences in sensitivity among aquatic invertebrate species range several orders of magnitude (Morrissey et al. 2015). More than two thirds of the data refer to imidacloprid, for which acute LC50s range from 4 μg/L in the most susceptible insect orders (Ephemeroptera, Trichoptera, and Diptera) to values exceeding 44,000 μg/L in the most tolerant cladoceran crustaceans. It is unfortunate that the standard species used in regulatory assessments, namely *Daphnia magna*, has a typical LC50 of about 100,000 μg/L, and this caused regulators to underestimate risks for some time. The study recommended ecological thresholds for neonicotinoids in water at concentrations below 0.2 μg/L for short-term acute exposures and 0.035 μg/L for long-term chronic exposures to avoid ecological impacts on aquatic invertebrate communities (Morrissey et al. 2015). Some of these impacts were described in a previous review (Pisa et al. 2015), and links between the toxic effects at the individual species, populations, and communities levels with impacts on aquatic and terrestrial ecosystems have been published more recently (Sánchez-Bayo et al. 2016a).

The decline of dragonflies and damselflies (Odonata) in Japan since the early 1990s has been blamed on the introduction of systemic insecticides in that country (Jinguji and Uéda 2015), but solid evidence was lacking. Impacts of these insecticides applied to rice seedlings in nursery-boxes at recommended rates were tested on the dragonfly *Sympetrum frequens*: none of the 50 nymphs introduced in experimental lysimeters treated with either imidacloprid or fipronil survived after 1 month, and only 13% remained in those treated with dinotefuran. Although the rate of adult emergence in the dinotefuran treatment was similar to that in the controls, the average head width of the dragonflies in this treatment was significantly narrower (Jinguji and Uéda 2015). In a similar study using paddy mesocosms treated with either clothianidin, fipronil, or chlorantraniliprole, the abundance of Odonata species in the clothianidin and specially in the fipronil-treated paddies was very low compared to controls and the other treatment (Kasai et al. 2016). Plankton species also declined in the clothianidin and chlorantraniliprole treatments right after the applications, but they recovered when their initial concentrations decreased to minimal levels. Previous mesocosm studies in Japan had showed the toxicity of imidacloprid to Odonata species when applied at the recommended rates to rice paddies (10 kg/ha). However, the study by Kobashi et al. (2017) also showed compensation among the predatory insects: while populations of *Crocothemis servilia mariannae* and *Lyriothemis pachygastra* nymphs were significantly reduced, those of *Orthetrum albistylum speciosum* increased slightly throughout the 5-month experimental period. Large decreases in the abundance of a common predator, *Notonecta triguttata*, were also observed, and the *Guignotus japonicus* disappeared, the effects on both species resulting from a delayed but measurable chronic toxicity (Kobashi et al. 2017). Other authors have determined the 48-h LC50 for clothianidin in North American dragonflies in the range 865 to 1245 μg/L (Miles et al. 2017).

Mayflies (Ephemeroptera) comprise other insect taxa very susceptible to neonicotinoids. The acute and chronic toxicity (28 days exposure) of thiamethoxam, thiacloprid, and imidacloprid to *Cloeon dipterum*, and their seasonal variability was studied by van den Brink et al. (2016). Thiacloprid was twice as toxic to the winter generation as the other two neonicotinoids, whereas both acute and chronic toxicity of imidacloprid to the summer generation was much higher than to the winter one. Camp and Buchwalter (2016) demonstrated that the higher susceptibility of 6 species of aquatic insects to imidacloprid during summer is due to the higher water temperatures during that season: the time-to-effect for sublethal impairment and immobility was significantly decreased with increasing temperature from 15 to 25 °C because the intake of the toxicant and metabolism also increased accordingly. For *Cloeon dipterum*, lethal median concentrations (LC50s) of the studied neonicotinoids (thiamethoxam, thiacloprid, and imidacloprid) decreased by a factor of 3 to 6 times between 24 and 96 h of exposure in either season (van den Brink et al. 2016). The same result was found with the lotic mayfly *Isonychia bicolor* exposed to imidacloprid (Camp and Buchwalter 2016). Moreover, chronic exposures of *C. dipterum* resulted in LC50s of 0.30 μg/L for thiacloprid, 0.32 μg/L for imidacloprid and 0.8 μg/L for thiamethoxam, the latter LC50s being 270, 800 and 100 times lower than their respective ones at 24 h (van den Brink et al. 2016). Also, LC50s for acute exposure of the freshwater amphipod *Gammarus kischineffensis* to thiamethoxam dropped from 75.6 μg/L at 24 h to 3.7 μg/L at 96 h, that is a 20-fold decrease in concentration in 4 days to achieve the same mortality effect (Uğurlu et al. 2015). These studies confirm the delayed and extreme chronic toxicity of neonicotinoids to aquatic organisms.

New toxicity data of aquatic predatory insects are now available for clothianidin (Miles et al. 2017). The 48-h LC50 for the aquatic beetle *Graphoderus fascicollis* (Dytiscidae) was determined at 2 μg/L, which indicates the susceptibility of this species compared to that of four species of water bugs (LC50 range 56–805 μg/L) and three species of dragonflies (LC50 range 865–1245 μg/L). The water bug *Belostoma flumineum* displayed a dose-dependent reduction in feeding rate after exposure to sublethal concentrations of clothianidin. The authors also carried out a mesocosm study to investigate the effect of three concentrations of clothianidin (0.6, 5, and 352 μg/L) in the arthropod communities. Predatory invertebrates experienced significant mortality with increasing levels of the insecticide in water, concomitant with increases of their prey up to 50% at the highest concentration, indicating a top-down trophic cascade in community abundance (Miles et al. 2017).

In laboratory tests with larvae of *Chironomus dilutus*, the 14-day LC50s for imidacloprid, clothianidin, and thiamethoxam were 1.52, 2.41, and 23.60 μg/L, respectively. However, the 40-d EC50s for adult emergence under exposure to the same chemicals were 0.39, 0.28, and 4.13 μg/L, respectively. This indicates that sublethal concentrations that prevent emergence of this key wetland species are between 4 and 9 times lower than those that cause mortality to the larvae (Cavallaro et al. 2017). Exposure of *Chironomus riparius* larvae to various mixtures of pyrethroid (deltamethrin and esfenvalerate) and neonicotinoid insecticides (imidacloprid and thiacloprid) at 50% of their known LC50s showed sometimes additive and other times antagonistic effects on survival (Kunce et al. 2015). In the case of the amphipod *Hyalella azteca*, combined exposure to imidacloprid and cyfluthrin resulted in mortality ratios of 1.7 to 2.7 higher than either insecticide alone, indicating greater than additive toxicity (Lanteigne et al. 2015).

Available toxicity data for amphipods indicate that these detritivores of organic material are less susceptible to neonicotinoids than insect larvae by one order of magnitude or more (Morrissey et al. 2015). However, such differences tend to be species specific. For example, recent studies have shown that the amphipod *Gammarus fossarum* is more susceptible than the caddisfly *Chaetopteryx villosa* when exposed to three neonicotinoids (imidacloprid, thiacloprid, and acetamiprid), either alone or in mixtures (Englert et al. 2017). Furthermore, the same study found that combined exposure of these shredder species to neonicotinoid residues in water and in food (tree leaves) had more negative impacts on their survival than direct exposure to contaminated water alone. Exposure of benthic organisms to residual neonicotinoids in water is already widespread in Europe: 47% of the 19 amphipods (*Dikerogammarus* spp.) collected from the Danube river in eastern Germany had residues of thiacloprid at levels 0.1–0.39 ppb (wet body weight) (Inostroza et al. 2016).

Mollusks are known to be quite tolerant of neonicotinoids (Morrissey et al. 2015), with water snails (*Physa acuta* and *Helisoma trivolvis*) having no mortality after exposure to 327 mg/L clothianidin for 2 days (Miles et al. 2017). It appears that the mechanism of toxicity of these insecticides on these organisms is different, as neonicotinoids inhibit the nACh receptors in the pond snail (*Lymnaea stagnalis*) instead of acting agonistically (Vehovszky et al. 2015). At environmentally relevant concentrations (0.1 to 100 μg/L), imidacloprid downregulated the production of many fatty acids in the snails, while the levels of polyamines, spermidine and putrescine increased, indicating neuron cell injury. Cholinergic gene expression was also increased as the snails tried to overcome imidacloprid binding to the nAChRs (Tufi et al. 2015). Exposure of the snails to mixtures of neonicotinoids and other pesticides found in water from agricultural areas in the Netherlands showed more disturbed metabolic pathways than exposures to individual chemicals (Tufi et al. 2016). Toxicity tests of a suite of neonicotinoids to the Ramshorn snail (*Planorbella pilsbryi*) showed 7-day LC50s of ≥ 4000 μg/L for imidacloprid, clothianidin, and thiamethoxam, respectively, whereas the 28-day LC50s were ≥ 182 μg/L for all three insecticides. However, growth and biomass are more sensitive endpoints than mortality for this species, with EC50s ranging from 33.2 to 122.0 μg/L (Prosser et al. 2016). Similarly, the 48-h LC50s for glochidia of the wavy-rayed lampmussel (*Lampsilis fasciola*) were ≥ 456 μg/L for all seven neonicotinoids tested by these authors. Thus, the aquatic studies to date confirm that neonicotinoids pose less of a hazard to mollusks compared to non-target aquatic insects.

Despite the above findings, a probabilistic risk assessment that compared the available acute and chronic toxicity data of neonicotinoids to the current levels of contamination of these insecticides in surface waters of the USA, concluded that “the aquatic invertebrate community is unlikely to be adversely affected by acute or chronic exposure to imidacloprid resulting from currently registered uses of imidacloprid in the United States” (Aslund et al. 2017). The study was funded by Bayer CropScience and three private environmental consulting firms. By contrast, a review of 29 surveys in nine countries found “that 81% (22/27) and 74% (14/19) of global surface water studies reporting maximum and average individual neonicotinoid concentrations respectively, exceeded the thresholds of 0.2 and 0.035 μg/L” for protection of 95% of aquatic species (Morrissey et al. 2015).

### The delayed mortality and chronic toxicity of neonicotinoids

**Fig.2:**
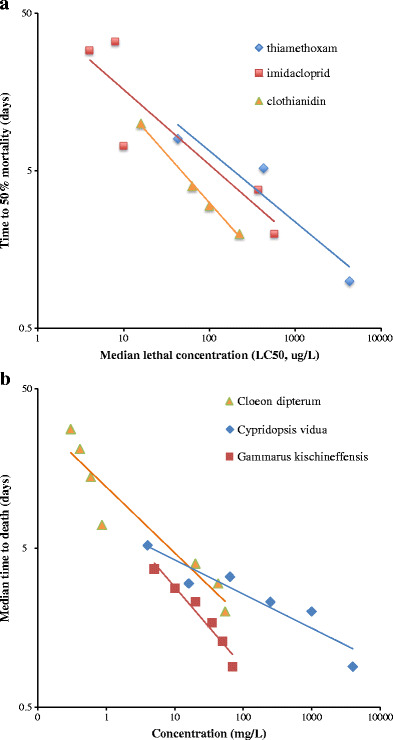
Time-cumulative toxicity of neonicotinoids in **a** *Apis mellifera* and **b** aquatic arthropods. Data sources: **a** clothianidin (*r* ^2^ = 0.99), Alkassab and Kirchner 2016; imidacloprid (*r* ^2^ = 0.81), Suchail et al. 2001 and Dechaume-Moncharmont et al. 2003; thiamethoxam (*r* ^2^ = 0.90), Oliveira et al. 2013; **b** *Cloeon dipterum* and thiacloprid (*r* ^2^ = 0.90), van den Brink et al. 2016; *Cypridopsis vidua* and imidacloprid (*r* ^2^ = 0.88), Sánchez-Bayo 2009; *Gammarus kischineffensis* and thiamethoxam (*r* ^2^ = 0.95), Uğurlu et al. 2015

Previous studies on the toxicity of neonicotinoids pointed to an increasing rate of mortality of the organisms exposed over time (Tennekes 2010; Tennekes and Sánchez-Bayo 2012), which results in lower LC50s under a continuous exposure to very low concentrations of these insecticides. As a result, the acute/chronic ratios span two or three orders of magnitude. The data available at that time referred to imidacloprid and thiacloprid and comprised only aquatic organisms, mainly insects and crustaceans. In recent years, two other studies about the chronic exposure of insect larvae (van den Brink et al. 2016) and gammarid amphipods (Uğurlu et al. 2015) have confirmed that, in addition, chronic exposure to thiamethoxam produces the same pattern of toxicity (Fig. 2a). The consequence is an apparent “delayed mortality” (Beketov and Liess 2008), which can be observed in mesocosm trials that use a single pulse exposure: most of the organisms do not die immediately but start dying in large numbers after a week, and their populations disappear completely after a few weeks (Hayasaka et al. 2012; Sánchez-Bayo and Goka 2006).

Delayed mortality was also demonstrated in honeybees exposed chronically to low concentrations of imidacloprid in syrup (Rondeau et al. 2014) and has now been confirmed with honey bees exposed to thiamethoxam (Oliveira et al. 2013) and clothianidin (Alkassab and Kirchner 2016) in their food (Fig. 2b). While the exposure of terrestrial organisms to insecticide residues in food is not constant, unlike the aquatic organisms that take up the toxicant through the gills, increased lethal effects over time in both cases suggest the same mechanism of toxicity applies to terrestrial and aquatic invertebrates alike.

The proposed mechanism for delayed mortality involves an irreversible binding to the nicotinic acetylcholine receptors (nAChR) embedded in the synaptic membranes of neurons, whose activation elicits a continuous electric impulse that eventually leads to the death of the neuron. The neuronal death toll accumulates as more and more pesticide molecules bind to other nAChRs until the organism cannot cope with the damage and dies. The main difference between this mode of action and that of other pesticides is that effects are cumulative with time, because neurons do not regenerate—it has been termed time-cumulative or reinforced toxicity (Tennekes and Sánchez-Bayo 2013). This mechanism certainly applies to all arthropods tested to date, but not to birds. Thus, while red-legged partridges that fed on wheat seeds coated with imidacloprid died within 3 weeks (Lopez-Antia et al. 2015a), their mortality did not follow the time-cumulative pattern, most likely because the binding of neonicotinoids to the subunits that make the nAChR in vertebrates is not as strong as in invertebrates (Tomizawa and Casida 2003).

The consequences of this particular pattern of chronic toxicity are far reaching. First, it indicates that acute LC50s or LD50s determined for short exposures (24 or 48 h) are irrelevant for risk assessments of these chemicals, because it is the long exposure to much lower levels of insecticide that really affects the survival of the organisms. It follows that protective levels for neonicotinoids cannot be achieved by setting a concentration benchmark, because the effects of neonicotinoids increase with exposure time and because of cascade effects within individuals. Second, when residues are present in the environment, constant or repeated exposure to any concentration of the insecticides will eventually produce large mortality rates in populations of susceptible invertebrates, e.g., soil-dwelling arthropods such as the grubs of many insects, aquatic arthropods, or pollinators exposed to low residue levels in nectar, pollen, and water. This time-cumulative effect may therefore be part of the explanation for the continuous decline of entire populations of insects that has been observed in Europe in recent decades (Sorg et al. 2013; Vogel 2017), thus reducing the food resources of songbirds (Hallmann et al. 2014) and probably other insectivorous animals such as shrews, lizards, and frogs. Consequently, the environmental impacts of neonicotinoids are not restricted to their direct toxic effects on insects but may reach beyond to the entire ecosystem, by indirectly impacting vertebrate populations that depend on arthropod resources for food.

## Part B: vertebrates

To update the assessment on the impacts of neonicotinoids and fipronil on vertebrates (Gibbons et al. 2015), a literature search was undertaken, using the same methods, but restricted to the years 2014–2016 and a few months in early 2017). Only sources not included in Gibbons et al. (2015) are presented here. In some cases, individual studies covered more than one species, chemical, or dosage approach (e.g., chronic or acute), and in all but three cases, which were field studies covering multiple bird or reptile species, each is treated here as a separate impact study.

As in the previous assessment, most studies were laboratory-based (111/129, 86%) and were of direct toxicity (124/129, 96%). This over-reliance on laboratory direct toxicity studies, as well as lack of field-based studies that can also identify indirect effects (e.g., cascades through the food chain) continues to limit our ability to interpret the findings under field-realistic conditions. Once again, the most common study taxa were mammals (51), fish (38), and birds (31), with substantially fewer studies of amphibians (6) and reptiles (3). Just over half of these studies were of five species: rat *Rattus norvegicus* (32 studies), mouse *Mus musculus* (14), zebrafish *Danio rerio* (8), rohu *Labio rohita* (7), and domestic chicken *Gallus gallus domesticus* (7).

In Gibbons et al. (2015), more than a third of all studies found (51/152) were of acute toxicity, measuring either LD50 or LC50_._ Over the period of this update only 13 studies (10%) explicitly measured LD50 or LC50, suggesting a changing focus among researchers to identify the sublethal, rather than lethal effects of these systemic insecticides. Nearly three-quarters of all studies investigated the effects of either the neonicotinoid imidacloprid (57) or fipronil (36). Given the paucity of information collated for the other neonicotinoids, this update largely concentrates on these two products, although it also includes clothianidin for comparability with the earlier review.

### Acute toxicity studies

Despite studies focusing mainly on sublethal effects, this updated review found imidacloprid LC50 values for six new species; rohu 550 mg/L (Qadir et al. 2015), white sturgeon *Acipenser transmontanus* 124 mg/L (Frew and Grue 2015), common carp *Cyprinus carpio* 78 mg/L for eggs and 280 mg/L for adults (Tyor 2016), loach *Misgurnus anguillicaudatus* 145.8 mg/L (Xia et al. 2016), Montevideo tree frog *Hypsiboas pulchellus* 84.9 mg/L (de Arcaute et al. 2014) and 52.6 mg/L for a formulation of imidacloprid, Glacoxan Imida (Pérez-Iglesias et al. 2014), and an LC50 value for zebrafish larvae 143.7 mg/L (Wang et al. 2016a). Following the classification of the US EPA (see Table 1, Gibbons et al. 2015), imidacloprid is practically non-toxic to the fish species studied—though slightly toxic to common carp eggs—and slightly toxic to Montevideo tree frogs, making it the most sensitive frog species studied.

Two additional fipronil LC50 values were also found: zebrafish larvae (0.6 mg/L; Yan et al. 2016) and common carp (0.43 mg/L; Qureshi et al. 2016). Fipronil is thus highly toxic to both these fish species, further confirming the toxicity of this chemical to fish (Wagner et al. 2017).

### Sublethal effects

Adding to the results in Gibbons et al. (2015), a further wide range of sublethal effects of imidacloprid, clothianidin and fipronil has been found under laboratory conditions (Table 3). In red-legged partridges *Alectoris rufa*, reproductive effects included reduced clutch size, delayed laying dates and altered secondary sexual characteristics when exposed to imidacloprid (Lopez-Antia et al. 2015a). Numbers of germ cells were reduced when Japanese quail *Coturnix japonica* were exposed to clothianidin (Hoshi et al. 2014), while exposure of red-legged partridges to fipronil led to reductions in the levels of sex hormones, reduced egg fertility, and altered secondary sexual characteristics (Lopez-Antia et al. 2015b). Imidacloprid and fipronil had deleterious effects on growth of birds, exhibited as growth retardation, reduced weight gain and lost body condition, often as a consequence of reduced food intake (Hussein et al. 2014a, b; Khalil et al. 2017; Lopez-Antia et al. 2015b). During embryonic development of birds, exposure to imidacloprid caused heart malformation (Gao et al. 2016), neural tube defects (Liu et al. 2016; Wang et al. 2016b), altered organ mass, and other anatomical deformities (Gobeli et al. 2017). In red-legged partridges, exposure to imidacloprid led to a depressed immune response in offspring of treated parents (Lopez-Antia et al. 2015a) and of parents and offspring when exposed to fipronil (Lopez-Antia et al. 2015b).

**Table3:** Sub-lethal direct effects of imidacloprid, clothianidin and fipronil on vertebrates from laboratory-based studies.Studies included in Gibbons et al. 2015 and 2016 are not repeated here. Dosages used in each study are given. Dosage could either be acute or chronic, the latter shown as /d (per day). All studies demonstrated deleterious effects at the given dosage, except those marked NE (no effect)

Taxon and species	Effect on:	Imidacloprid	Clothianidin	Fipronil	Source and detailed effect
Mammal					
Rat, *Rattus norvegicus*	Reproduction	38 mg/kg/d ^a^		0.1 mg/kg/d (NE) ^b^ 0.03–3 mg/kg/d ^c^	^a^ Lohiya et al. 2016; increased zinc uptake in ovaries may affect synthesis of reproductive hormones ^b^ Udo et al. 2014; no effect on gestation or reproductive quality ^c^ de Barros et al. 2016; perinatal exposure led to reduction in sperm mobility, though no other effects on reproduction
Rat, *Rattus norvegicus*	Growth and development			30 mg/kg/d	Chaguri et al. 2016; reduced weight gain
Rat, *Rattus norvegicus*	Genotoxic	0.26 mg/L ^a^ 170 mg/kg ^b^	24 mg/kg/d(NE) ^c^		^a^ Kimura-Kuroda et al. 2016; several genes essential for brain development were up or down regulated at these chronic low doses ^b^ Arslan et al. 2016; sex-specific genotoxicity at LD_50_ dose; males more prone to genotoxicity ^C^ Ozdemir et al. 2014; no impact detected on expression of genes in the hippocampus
Rat, *Rattus norvegicus*	Cytotoxic	1.1, 4, 20, 20, 40 mg/kg/d ^a,b,c,d,e^ 170 mg/kg ^f^	12 mg/kg/d ^g^	5, 24, 30 mg/kg/d ^h,i,j^ 4.85 mg/kg/3.5d ^k^ 2.19, 10.9 mg/L ^l,m^	^a^ Ibrahim et al. 2015; disruption of thyroid hormone levels ^b^ Ozsahin et al. 2014; biochemical alterations in kidneys ^c^ Kapoor et al. 2014; increased levels of serum enzymes ^d^ Vohra et al. 2014; marked changes to liver tissue and degeneration of hepatocytes ^e^ Annabi and Dhouib 2015; alteration of biochemical processes in the hypothalamic-adrenal-pituitary tissues ^f^ Arslan et al. 2016; sex-specific cytotoxicity at LD_50_ dose ^g^ Ozsahin et al. 2014; biochemical alterations in kidneys ^h^ Caballero et al. 2015; induces cytochrome P450 enzyme activity in liver microsomes ^i^ Kartheek and David 2016; oxidative stress and structural damage to liver ^j^ Chaguri et al. 2016; increased systolic blood pressure causing hypertension ^k^ Ehsan et al. 2016; thyroid damage causing thyroid hormone changes ^l^Tavares et al. 2015a, b; impacts on liver mitochondrial bioenergetics ^m^ Guelfi et al. 2015; inhibition of mitchondrial activity leading to hepatocyte death
Rat, *Rattus norvegicus*	Neurobehavioural	2 mg/kg/d ^a^	24 mg/kg/d ^b^	0.1, 0.1, 1, 30 mg/kg/d ^c,d,e,f^	^a^ Kara et al. 2015; learning activities diminshed in infants ^b^ Ozdemir et al. 2014; deterioration in cognitive function (learning and memory) in infant, though not adult rats ^c^ Magalhaeset al. 2015; disturbed maternal aggressive behavior against male intruders ^d^ Udo et al. 2014; disturbed maternal behavior and reflex development in offspring ^e^ Montanha et al. 2016; lactational exposure led to impaired memory in offspring ^f^ Godinho et al. 2017; memory impairment
Mouse, *Mus musculus*	Reproduction		250 mg/kg/d (NE)		Hirano et al. 2015; no effect on testes weight
Mouse, *Mus musculus*	Growth and development	0.5, 15 mg/kg/d ^a,b^	250 mg/kg/d ^c^		^a^ Burke 2016; in utero and post natal exposure reduced body weight ^b^ Arfat et al. 2014; body weight reduced ^c^ Hirano et al. 2015; body weight reduced
Mouse, *Mus musculus*	Genotoxic	4.5 (NE), 22 mg/kg/d ^a,b^ 75 mg/kg ^c^	20 mg/kg/3d ^d^	9.5 mg/kg ^e^	^a^ Saxena and Kesari 2016; no change in frequency of chromosomal aberrations and micronuclei frequency ^b^ Bagri et al. 2016; increased chromosomal aberrations and micronuclei frequency in somatic cells ^c^ Kataria et al. 2016; induced mitotic inhibition; at 112.5 mg/kg caused micronuclear formation ^d^ Calderon-Segura et al. 2015; DNA damaged in peripheral blood cells, and increase in micronuclei frequency in the peripheral blood erythrocytes ^e^ Lovinskaya et al. 2014; increase in chromosomal aberrations in bone marrow cells
Mouse, *Mus musculus*	Cytotoxic	15 mg/kg/d ^a^ 35, 112.5 mg/kg ^b,c^			^a^Arfat et al. 2014; hepatotoxicity and nephrotoxicity induced ^b^Kumar et al. 2014; toxic effects on both biochemical and histological parameters found ^c^ Kataria et al. 2016; changes in red and white blood cells, and hemoglobin and erythrocyte sedimentation rates
Mouse, *Mus musculus*	Neurobehavioural	0.5 mg/kg/d ^a^	10 mg/kg/d ^b^		^a^ Burke2016; in utero and post natal exposure caused increased motor activity and social dominance, and reduced depressive behavior and aggression ^b^ Hirano et al. 2015; anxiety-like behaviors increased, especially when stressed
Chinese hamster, *Cricetulus griseus*	Genotoxic	29 mg/L			Al-Sarar et al. 2015; genotoxic effects in ovary cells, specifically induction of micronuclei
Rabbit, *Oryctolagus cuniculus*	Genotoxic	40 mg/kg/d			Stivaktakis et al. 2016; increased frequency of micronuclei
Rabbit, *Oryctolagus cuniculus*	Cytotoxic	40 mg/kg/d (NE)			Stivaktakis et al. 2016; no cytotoxic effects observed
Formosan leaf-nosed bat, Hipposideros terasensis	Neurobehavioural	20 mg/kg/d			Hsiao et al. 2016; spatial memory disruption in echo-locating bats
Bird					
Chicken, *Gallus gallus domesticus*	Growth and development	128, 128, 16 mg/L ^a,b,c^ 0.025 mg ^d^			^a^ Gao et al. 2016; malformation of heart during embryonic development ^b^ Wang et al. 2016b; embryonic neural crest cell development disrupted ^c^ Liu et al. 2016; neural tube defects during embryo development ^d^ Hussein et al. 2014a, b; growth retardation of developing embryos and knock-on effects. (NB: not corrected for body weight)
Chicken, *Gallus gallus domesticus*	Cytotoxic	1/20th LD50			Tiwari et al. 2016; liver degeneration, necrosis of hepatocytes and disruption of hepatic cord. (NB: dosage not given)
Red-legged partridge, *Alectoris rufa*	Reproduction	8.8 mg/kg/d ^a^*		8.73 mg/kg ^b^**	^a^ Lopez-Antia et al. 2015a;causes reduced clutch size, delayed first egg date and changes in eye ring colouration (a secondary sexual trait) ^b^Lopez-Antia et al. 2015b; reduced egg fertility, which could have been caused by observed reduction in sex hormones of treated males; also reduced carotenoid pigmentation in eye ring, a sexual ornament
Red-legged partridge, *Alectoris rufa*	Growth and development			8.73 mg/kg **	Lopez-Antia et al. 2015b; reduced food intake and lost body condition
Red-legged partridge, *Alectoris rufa*	Cytotoxic	8.8 mg/kg/d ^a^*		8.73 mg/kg ^b^**	^a^ Lopez-Antia et al. 2015a; reduced levels of plasma biochemistry parameters and increased blood superoxide dismutase activity ^b^Lopez-Antia et al. 2015b; reduced antioxidant levels in adults and eggs
Red-legged partridge, *Alectoris rufa*	Immunotoxic	8.8 mg/kg/d ^a^*		8.73 mg/kg ^b^**	^a^Lopez-Antia et al. 2015a; depressed T cell immune response in chicks of treated parents ^b^Lopez-Antia et al. 2015b; reduced cellular immune response in adults and offspring
Japanese quail, *Coturnix japonica*	Reproduction		0.1 mg/kg/d ^a^	2.26 mg/kg/d ^b^	^a^ Hoshi et al. (2014); decreased number of germ cells, but little effect on egg weights or fertilization rates of females bred with treated males ^b^ Khalil et al. 2017; estrogenic activity in males that can cause sterility
Japanese quail, *Coturnix japonica*	Growth and development			2.26 mg/kg/d	Khalil et al. 2017; loss in feed rate and weight
Japanese quail, *Coturnix japonica*	Genotoxic		0.1 mg/kg/d ^a^	2.26 mg/kg/d ^b^	^a^ Hoshi et al. (2014); increased DNA fragmentation in germ cells of seminiferous epithelium ^b^ Khalil et al. 2017; alterations to estrogen receptor α gene expression
Japanese quail, *Coturnix japonica*	Cytotoxic			2.26 mg/kg/d	Ali et al. 2016; biochemical and histopathological changes confirming potentially hepatotoxic and somewhat nephrotoxic impact
Japanese quail, *Coturnix japonica*	Neurobehavioural			2.26 mg/kg/d	Khalil et al. 2017; altered sexual behavior
Bob-white quail, *Colinus virginianus*	Growth and development	150 mg/kg			Gobeli et al. 2017; anatomical deformities and altered organ mass in embryos
Red munia, *Amandava amandava*	Cytotoxic	0.16 mg/kg/d			Pandey and Mohanty 2015; disruption of thyroid physiology, which could ultimately have reproductive consequences
Fish					
Zebrafish, *Danio rerio*	Growth and development			0.1 mg/L	Yan et al. (2016); uninflated swim bladder in embryos; reduced body length at 0.2 mg/L and bent spine at 0.4 mg/L
Zebrafish, *Danio rerio*	Genotoxic	1.25 mg/L			Ge et al. 2015; induced DNA damage
Zebrafish, *Danio rerio*	Cytotoxic	1.25 mg/L			Ge et al. 2015; induced oxidative stress
Zebrafish, *Danio rerio*	Neurobehavioural	11.5 mg/L ^a^		0.01–0.04 mg/L ^b^	^a^ Crosby et al. 2015; reduced swimming activity in larvae, and reduced novel tank exploration and increased response to startle stimuli in adolescents and adults. ^b^ Wang et al. (2016a); anxiety-like behavior, including increased swimming speed, and abnormal photoperiod accommodation (at 0.04 mg/L) in larvae
Nile tilapia, *Oreochromis niloticus*	Genotoxic	0.0625 mg/L			Ansoar-Rodríguez et al. 2015; primary damage to DNA
Nile tilapia, *Oreochromis niloticus*	Cytotoxic	0.0625 mg/L ^a^		0.002 mg/L ^b^	^a^ Ansoar-Rodríguez et al. 2016; histopathological changes in the liver and active defense mechanism to maintain liver integrity ^b^ El-Murr et al. 2015; a reduction in erythrocyte and leucocyte count, and hemoglobin content, and a range of other histopathological and biochemical effects
Nile tilapia, *Oreochromis niloticus*	Immunotoxic			0.002 mg/L	El-Murr et al. 2015; reduction in level of Immunoglobulin M and lysozyme
Medaka, *Oryzias latipes*	Reproduction			0.003, 0.2 mg/L ^a,b^	^a^ Sun et al. 2014; changes to the hypothalamic-pituitary-gonadal axis ^b^ Wagner et al. 2017; reduced hatching success, with delayed hatching at 0.6 mg/L
Medaka, *Oryzias latipes*	Growth and development			0.003, 0.2 mg/L ^a,b^	^a^ Sun et al. 2014; inhibited growth in larvae of both sexes ^b^ Wagner et al. 2017; increased gross deformities (tail curvature)
Rohu, *Labio rohita*	Growth and development	120 mg/L			Qadir et al. 2015; reduced body weight after long-term exposure
Rohu*, Labio rohita*	Cytotoxic	120 mg/L			Qadir et al. 2014; anemia and disturbance of liver physiology Qadir and Iqbal (2016); severe degenerative changes to liver; no changes to heart
White sturgeon*, Acipenser transmontanus*		0.7 mg/L (NE)			Frew and Grue 2015; no observed adverse effects
Common carp, *Cyprinus carpio*	Reproduction	7.8 mg/L			Tyor (2016); 10% of LC50 reduced egg hatchability
Common carp, *Cyprinus carpio*	Genotoxic			0.4 mg/L	Qureshi et al. 2016; exposure induced genetic damage
Common carp, *Cyprinus carpio*	Cytotoxic			0.00065, 0.043, 0.4 mg/L ^a,b,c^	^a^ Menezes et al. 2016; oxidative damage and hyperglycemia ^b^ El-Murr et al. 2016; hepatotoxic, e.g., decreased hepatic antioxidant activities ^c^ Qureshi et al. 2016; biochemical, hematological, and histopathological damage
Silver catfish, *Rhamdia quelen*	Cytotoxic			0.00065	Menezes et al. 2016; oxidative damage and hyperglycemia
Slender rasbora, *Rasbora danonicus*	Cytotoxic	0.8 mg/L			Gaikwad and Reddy 2016; decreased rate of oxygen consumption
Chameleon cichlid, *Australoheros facetus*	Genotoxic	0.1 mg/L			Iturburu et al. 2017; increased frequency of micronuclei
Amphibia					
Montevideo tree frog, *Hypsiboas pulchellus*	Genotoxic	15, 25 mg/L ^a,b^			^a^ de Arcaute et al. 2014; induced DNA damage leading to micronucleotide formationand other nuclear anomalies ^b^ Perez-Iglesias et al. 2014; induced DNA damage leading to micronucleotide formation
Cuyaba dwarf frog, *Eupemphix nattereri*	Cytotoxic			0.035 mg/kg	Gripp et al. 2017; oxidative stress, i.e., decreased catalase activity and increased lipid peroxidation at environmentally relevant levels (e.g., when pools dry up)
Reptile					
Italian wall lizard, *Podarcis sicula*	Reproduction	10 mg/kg/2d			Cardone 2015; decrease both the level of sex hormones and the steroid receptor mRNAs

*Wheat seeds treated with 20% of manufacturer’s recommended rate

**Maize seeds treated with manufacturer’s recommended rate

All three chemicals caused a wide range of cytotoxic effects in vertebrates (see Table 3 for references), including disruption of thyroid hormones and thyroid physiology, alterations to red and white blood cells, hepatotoxicity and nephrotoxicity, and induction of oxidative stress (imidacloprid); disrupted kidney biochemistry (clothianidin); structural damage and histopathological changes in the liver, thyroid damage, oxidative stress and hyperglycemia, and inhibition of mitochondrial activity leading to hepatocyte death (fipronil).

In mammals, neurobehavioral effects included diminished learning ability (Kara et al. 2015), enhanced social dominance and reduced aggression (Burke 2016), and disrupted spatial memory during echo-location in bats when exposed to imidacloprid (Hsiao et al. 2016); deteriorated cognitive function in infants (Ozdemir et al. 2014) and increased anxiety-like behavior (Hirano et al. 2015) when exposed to clothianidin, and impaired memory following lactational exposure (Montanha et al. 2016), disturbed maternal behaviors, such as aggression (Magalhaes et al. 2015), and altered reflex development in offspring (Udo et al. 2014) when exposed to fipronil. Exposure to imidacloprid also altered the regulation of genes important in mammalian brain development (Kimura-Kuroda et al. 2016).

In zebrafish, exposure to fipronil stopped swim bladders from inflating and caused curvature of the spine (Yan et al. 2016), while DNA damage and oxidative stress occurred following exposure to imidacloprid (Ge et al. 2015). Similar genotoxic and cytotoxic effects occurred in carp *Cyprinus carpio* and silver catfish *Rhamdia quelen* exposed to fipronil (Qureshi et al. 2016; Menezes et al. 2016). Genotoxic effects were also observed in chameleon cichlids *Australoheros facetus* exposed to imidacloprid (Iturburu et al. 2017). Histopathological changes in the liver were observed in Nile tilapia *Oreochromis niloticus* after exposure to imidacloprid (Ansoar-Rodríguez et al. 2016), while exposure to fipronil caused a reduction in immune system parameters (El-Murr et al. 2015). Medaka fish *Oryzias latipes* exposed to fiponil hatched less successfully, grew less well, and suffered tail deformities (Sun et al. 2014; Wagner et al. 2017), while Rohu *Labio rohita* exposed to imidacloprid grew less well and became anemic (Qadir et al. 2014, 2015). By contrast, no apparent effects on the white sturgeon (*Acipenser transmontanus*) were observed after treatment with imidacloprid (Frew and Grue 2015).

Once again, many of these sublethal effects occurred at much lower concentrations than lethal effects (Table 3). Thus, for example, while rat LD50 values for imidacloprid, clothianidin, and fipronil are 425–475, 5000, and 97 mg/kg, respectively, cytotoxic effects were detected with daily doses of 1.1, 12, and 5 mg/kg, respectively, and neurobehavioral effects with daily doses of 2, 24, and 0.1 mg/kg, respectively (references given in Table 3). Similarly, while mouse LD50 values for imidacloprid, clothianidin and fipronil are 131–300, > 389, and 95 mg/kg, respectively, genotoxic effects were detected with daily doses of 22, 20, and 9.5 mg/kg, respectively, and neurobehavioral effects with daily doses of 0.5 (imidacloprid) and 10 (clothianidin) mg/kg (references given in Table 3). While a dose of 53 mg/kg/day of imidacloprid reduced survival of adult red-legged partridges, one-sixth of this (8.8 mg/kg/day) caused reproductive, cytotoxic and immunotoxic effects, in the latter case in the offspring of treated parents. Similarly, while the red-legged partridge LD50 for fipronil is 34 mg/kg, a quarter of that dose (8.7 mg/kg) caused reproductive, cytotoxic, and immunotoxic effects, the latter in both treated adults and their offspring (references given in Table 3). In some cases, sublethal effects occur at doses several orders of magnitude lower than lethal doses. Thus, an in utero and post-natal daily dose of 0.5 mg/kg of imidacloprid led to increased motor activity and social dominance, and reduced depressive behavior and aggression in mice (LD50 = 131–300 mg/kg), while a concentration of 0.65 μg/L of fipronil caused oxidative damage and hyperglycemia in the common carp (LC50 = 0.43 mg/L) (Table 3).

### Risks to vertebrates from direct toxicity

Morrissey et al. (2015) documented global mean average, and mean peak surface water concentrations of neonicotinoids of 0.13 and 0.63 μg/L, with concentrations of imidacloprid and clothianidin ranging from 0.001–320 and 0.003–3.1 μg/L, respectively. Imidacloprid LC50 values for fishes and amphibians (Table 1 in Gibbons et al. 2015 and here) range from 1200 to 550,000 μg/L and for clothianidin from 94,000 to 117,000 μg/L (Table 1 in Gibbons et al. 2015, fish only). Thus, except in the most extreme cases, these aquatic vertebrates are very unlikely to be exposed to lethal concentrations of these two neonicotinoids in their natural environment. However, the possibility of sublethal effects of imidacloprid cannot be ruled out, with immunotoxic effects recorded in fish at 30 μg/L (Table 2 in Gibbons et al. 2015), and cytotoxic and genotoxic effects at 60 μg/L (Table 3).

Recorded surface water concentrations of fipronil have been documented as ranging from 0.004–6.4 μg/L (Gibbons et al. 2015; Mize et al. 2008) and 0.13–12 μg/L (Gan et al. 2012) and are thus within an order of magnitude of LC50 values for some fish (e.g., for Nile tilapia, *Oreochromis niloticus*, of 42 μg/L) and encompass part of the range at which sublethal effects have been detected, from 0.2–400 μg/L (Table 3). Thus, this review further supports the assertion of Gibbons et al. (2015) that some recorded environmental concentrations of fipronil may be sufficiently high to harm fish.

The most likely route of exposure to high concentrations of neonicotinoids among terrestrial vertebrates is through the ingestion of treated seeds (Goulson 2013; Mineau and Palmer 2013). Since the previous review, further evidence has come to light that supports this view. A laboratory study has shown that adult female red-legged partridges fed solely on a diet of wheat seeds treated with imidacloprid at field-realistic (i.e., manufacturer’s recommended) rate, and equivalent to a dose of 44 mg/kg/day, were killed, on average, within 7 days; males, within 13 days (Lopez-Antia et al. 2015a). A wide range of sublethal effects (Table 3) were also recorded among birds fed on seeds treated with one-fifth of this rate. While red-legged partridges have been shown to avoid imidacloprid-treated seeds due to post-ingestion distress, poisoning still occurs even when alternative food sources are available (Lopez-Antia et al. 2014). Partridges fed on fipronil-treated maize seed, again at a field-realistic rate, suffered a range of sublethal effects (Table 3); they did not reject the treated seeds, rather reduced intake rate and lost body condition (Lopez-Antia et al. 2015b).

#### Studies in the natural environment

Laboratory studies suggest that neonicotinoids and fipronil can kill or harm vertebrates, and inferences can be made from laboratory studies adopting field-realistic conditions. But there are only a few studies of the impact of these systemic insecticides on vertebrate wildlife in their natural environment, involving environmentally relevant concentrations. While several were reviewed in Gibbons et al. (2015), others have been published since. Turaga et al. (2016) examined the crops of nearly 100 wild-caught quail (northern bobwhite, *Colinus virginianus*, and scaled, *Callipepla squamata*) but did not find any neonicotinoid-treated seeds; however, the authors note that this study was undertaken in an area with limited use of neonicotinoids. Bro et al. (2016) detected concentrations of up to 67 and 8.5 ppb of thiamethoxam/clothianidin and fipronil (plus fipronil sulfone), respectively, in grey partridge (*Perdix perdix*) eggs. While the impact of these concentrations on egg viability was unknown, they were probably too low (Table 2 in Gibbons et al. 2015; Table 3 here) to have had any deleterious effects. By contrast, Lopez-Antia et al. (2016) estimated a potential mean daily intake by individual red-legged partridges of 23.4 and 41.7 mg/kg of imidacloprid and fipronil, respectively, from eating treated seeds left on the surface at sowing. Such a daily dose of imidacloprid exceeds that which causes sublethal effects in red-legged partridges, while the dose of fipronil exceeds the species LD50. Millot et al. (2015) reported that about 10% of the grey partridges found in their study fields that were exposed to thiacloprid, subsequently died, even though few direct impacts of other pesticides were detected. Finally, Millot et al. (2017) suggested that in France, mortality due to poisoning by imidacloprid-treated seeds was at least likely in 70% of wildlife mortality incidents reported during 1994–2014. Lopez-Antia et al. (2016) conclude that the use of pesticide-treated seeds presents an unacceptable risk to farmland birds.

Several impacts other than from direct poisoning have been reported. In the Netherlands, Hallmann et al. (2014) found that local population trends of insectivorous birds were more negative in areas with higher surface water concentrations of imidacloprid; populations declined when concentrations exceeded 20 ng/L. While this study was correlative, rather than experimental, and took no account of co-occurring pesticides (Vijver and van den Brink 2014), the effects remained after correcting for other potential drivers of change in bird populations. Given that bird numbers declined at exceptionally low imidacloprid concentrations, the most likely impact of this neonicotinoid was indirect, by reducing their invertebrate food supply, although other mechanisms could not be ruled out.

Two further studies have examined the impact that fipronil sprayed to control either locusts (Maute et al. 2016) or yellow crazy ants, *Anoplolepis gracilipes* (Stork et al. 2014) had on reptile and bird populations. While no impact was detected on local populations of 23 reptile and 3 bird species, fipronil spraying led to a drop in numbers of one bird species (Christmas Island imperial pigeon, *Ducula whartoni*) and a short-term increase in another (Christmas Island white-eye, *Zosterops natali*). The rise in white-eye numbers was probably an indirect, food chain effect caused by a temporary increase in the number of moribund insects as prey. However, the pigeon being a frugivore, its decline may have been due to a direct toxic impact on its reproduction.

#### Summary of impacts on vertebrates

In the past 3 years, further evidence has highlighted negative impacts of the neonicotinoids imidacloprid and clothianidin, and fipronil (a phenyl pyrazole) on vertebrate wildlife. All three insecticides exert a wide range of deleterious sublethal effects in the laboratory, with imidacloprid, for example, altering the regulation of genes important in rat brain development and disrupting the spatial memory of echo-locating bats. These sublethal effects are often detected at concentrations substantially lower than those causing lethal effects. Except in the most extreme cases, however, aquatic vertebrates are very unlikely to be exposed to lethal concentrations of these two neonicotinoids in their natural environment, although sublethal effects of imidacloprid cannot be ruled out and is expected in some cases. By contrast, some recorded environmental concentrations of fipronil may be sufficiently high to harm fish.

Since the earlier review, new evidence has emerged suggesting that terrestrial vertebrates can be exposed to high concentrations of neonicotinoids by ingesting treated seeds left on the surface at sowing. One study estimated that the daily doses of insecticides ingested by red-legged partridges could cause sublethal (imidacloprid) or even lethal (fipronil) effects (Lopez-Antia et al. 2016). A second concluded that mortality due to poisoning by imidacloprid-treated seeds was at least likely in 70% of reported wildlife mortality incidents (Millot et al. 2017). Evidence of the indirect, food chain effects of these insecticides remains rare, though one correlative study found that some local populations of insectivorous birds declined more in areas with higher surface water concentrations of imidacloprid, possibly as a consequence of reduced invertebrate food supplies (Hallmann et al. 2014).

## Part C: Ecosystem services

Ecosystem services are defined as regulation of ecosystem processes (e.g., decomposition, carbon sequestration, pollination, water purification), provision of goods (e.g., timber, food, molecules of pharmaceutical value), habitat for biodiversity (including antagonists of agricultural pests), or other non-material characteristics (e.g., landscape integrity, cultural reference) that are considered as valuable either to human societies or to the environment (de Vries et al. 2013; Melathopoulos et al. 2015; Paetzold et al. 2010; Droz et al. 2009). There is broad scientific consensus that more biodiverse ecosystems generally provide more/better ecosystem services and that the services provided are more resistant to or resilient following stress and perturbation (Isbell et al. 2011; Worm et al. 2006). It follows that any factor that has a significant negative impact on biodiversity in general or specifically on key organisms responsible for providing valuable services (e.g., pollinators) will reduce the value of the services provided and in extreme cases cause the total loss of the service.

Systemic neonicotinoid and fipronil pesticides have been shown to impact non-target organisms and may subsequently affect several ecosystem services such as pollination (by impacting bees, butterflies and other pollinators), nutrient cycling (e.g., by impacting soil or aquatic microorganisms, earthworms, etc.), fish productivity (e.g., by impacting aquatic invertebrates), and agricultural production, if the negative effects on useful non-target organisms outweigh the positive effects on plant protection (Chagnon et al. 2015). However, assessing such impacts is much more challenging than determining thresholds of acute toxicity of each compound. Partly due to a stronger research focus on other environmental stressors, including climate change, the body of knowledge on the effect of systemic pesticides on ecosystem services is limited (Bernhardt et al. 2017).

Regarding impacts on ecosystem functioning and ecosystem services, the WIA study concluded that the large-scale bioavailability of neonicotinoids, in particular, but also of fipronil, in the global environment occurs at levels that are known to cause lethal and sublethal effects on a wide range of terrestrial (including soil) and aquatic microorganisms, invertebrates and vertebrates. Population-level impacts occur at observed environmental concentrations in the field for insect pollinators, soil invertebrates, and aquatic invertebrates, which subsequently impair ecosystem functioning and services (Van der Sluijs et al., 2015).

Since the publication of the WIA, the evidence has further strengthened that these effects impair ecosystem functioning, resilience, and the services and functions provided by terrestrial and aquatic ecosystems. We review here the relevant studies published since 2015 that either specifically dealt with the impact of these insecticides on ecosystem services (e.g., pollination) or that affect key ecosystem functions. We mainly focus on the progress regarding the knowledge gaps regarding impacts on ecosystem services identified in the WIA study, especially (a) impact of accumulation in soil and sediments on soil health, soil structure and permeability and nutrient cycling; (b) impacts on pollination, pest control services and fauna valued for esthetic reasons (e.g., butterflies); (c) impacts of depletion of farmland insect and aquatic insect populations on insectivorous species such as birds and bats; (d) (indirect) impacts of contamination of freshwater on insect-eating fish and subsequently fisheries and other insectivores such as amphibians; and (e) impacts on coastal marine systems such as coral reefs, and salt marsh estuaries.

### Impacts on the soil ecosystem

Since their introduction in the United States and Europe in the mid-1990s, the use of neonicotinoids has increased rapidly as seed-applied products were introduced in field crops (particularly in corn and soybean between 2011 and 13), marking an unprecedented shift toward large-scale, prophylactic insecticide use (Simon-Delso et al. 2015). This means that more and more agricultural land is being loaded with neonicotinoid residues every year, since only maximum 20% of the insecticide present in the coating of the seeds is taken up by the crop plants (Goulson 2013), the remainder being left in the soil of the field. The ecological consequences of this accumulation of contamination in the agricultural land have not been studied in detail yet, but a few recent studies provide some indication.

Soil enzymes are indicators of microbial activities in soil and can therefore be used as biomarkers of soil health and fertility. Jyot et al. (2015) tested the impact of cotton seeds coated with thiamethoxam at two different rates (standard application 2.1 g a.i./kg seed and a high rate of 8.4 g a.i./kg seed) on soil enzymes in Pakistan. The activities of dehydrogenase and phosphatase enzymes were significantly reduced in the soils treated at both rates, this effect being most pronounced after 15 or 21 days, whereas the activity of urease was not affected. Their findings suggests that microbial soil communities are depleted in the first 3 weeks after planting, although their recovery occurs after thiamethoxam residues in soil are reduced (Jyot et al. 2015).

In a mesocosm study, pots were treated with wheat-seed dressings containing imidacloprid and fungicides. Seed dressings increased the number of protozoa and reduced plant decomposition rate but did not affect earthworm activity. Fungicides, in particular, increased collembola surface activity, which in turn influenced the activity of earthworms (*Lumbricus terrestris*), but reduced soil basal respiration. Earthworms also decreased wheat growth, reduced the soil basal respiration and microbial biomass, but increased the soil water content and electrical conductivity (Zaller et al. 2016). However, in a similar experiment that used the same seed treatment, soil basal respiration, microbial biomass, and litter decomposition were not affected. In the latter study, seed dressings significantly reduced the surface activity of earthworms independently of whether fungicides were used or not. By contrast, earthworm activity was intensified by glyphosate herbicide applied to the pots (Van Hoesel et al. 2017).

While there is no evidence of bioaccumulation of neonicotinoids in organisms, the systemic insecticide fipronil appears to accumulate in earthworms. Chronic exposure of *Eisenia fetida* to sublethal levels (10 to 50 mg/kg soil) of a racemic mixture of fipronil for 28 days showed accumulation of this insecticide in the tissues, which appears to be enantioselective: the S-fipronil enantiomer and the fipronil sulfone metabolite were preferentially found in the tissues, mainly because the elimination rate of R-fipronil was higher than that of the S-enantiomer. Because of the relative lipophilicity of these compounds and their slow rate of depuration, the authors warned of potential bioaccumulation through the food chain (Qin et al. 2015).

One way of reducing the pesticide residue loads in soil is by using soil amendments. A microcosm that contained soil contaminated with imidacloprid was treated with two soil amendments (vine-shoot and olive cake) at different rates, and incubated for 3 months. The dissipation rate constant of imidacloprid correlated well with changes in the bacterial community during incubation in the contaminated soil amended with olive-vermicompost. The study suggested that amendment of imidacloprid-contaminated soil with this type of vermicompost can mitigate the impact of this insecticide on soil functions and promote its depuration capability, thus minimizing environmental risks to other soil organisms (Castillo Diaz et al. 2017). Schaafsma et al. (2016) suggest rotational crops to reduce the burden in soils treated with clothianidin- and thiamethoxam-coated seeds. Other authors have indicated that neonicotinoids could be more carefully targeted, considerably reducing use without adversely affecting yield, and with considerable benefits for reducing the potential for pest resistance, outbreaks of non-target pests, and overall harm to the environment (Douglas and Tooker 2015).

### Impacts on pollination services

Pollination is an essential regulating, supporting, and cultural ecosystem service that comprises an integrated system of interactions that links earth’s vegetation, wildlife, and human welfare (Kevan and Menzel 2012; Van der Sluijs and Vaage, 2016). Pollination is essential for the setting of fruits and seeds of many crops and wild plants. Up to 94% of all flowering plants on earth benefit from animal pollination for reproduction and evolution (IPBES 2016b; Van der Sluijs and Vaage 2016). Globally, 87 of humanity’s major food crops depend on animal pollination (Klein et al. 2007). These account for 35% of global crop production volume (IPBES 2016b) and include vegetable, fruit, nut, and edible oil and proteinaceous crops, as well as spices and condiments (Maxim and Van der Sluijs 2013). Many fiber and fodder crops also depend on insect pollination. Loss of insect pollinators can thus indirectly affect the production of livestock agriculture. Biofuel crops (e.g., canola) and timber production (trees) also require animalpollination. The majority ornamental flowering plants as well as plants used for production of phytopharmaceuticals also depend on pollinators. The quality (especially fruit quality), shelf life, and commercial value of crops also benefits from insect pollination (Klatt et al. 2014). It is also essential for the genetic diversity of wild flowering plants (Benadi et al. 2013).

Further, pollinator-mediated crops are of key importance in providing essential nutrients in the human diet. They account for more than 90% of vitamin C, 100% of lycopene and almost 100% of the antioxidants β-cryptoxanthin and β-tocopherol, the majority of the lipids (74%), vitamin A (> 70%) and related carotenoids (98%), calcium (58%) and fluoride (62%), and a large portion of folic acid (55%). In total, pollinator-mediated crops account for about 40% of global nutrient supply for humans (Eilers et al. 2011). At present, an estimated 2 billion people suffer from deficiencies of such micronutrients, also known as hidden hunger (IFPRI 2014; Nicole 2015).

Pollination is also essential for sustaining the diet of wildlife. Many bird and mammal species feed on wild fruit (e.g., birds that feed on all kinds of wild berries in forests), wild nuts, and seeds of wild plants. If all insect pollinators were removed, this would result in a drastic decline in setting of wild fruits, nuts, and seeds, which would affect all species that depend on it. Herbivores can also suffer from food depletion if pollinator-dependent plants on which these herbivores depend can no longer reproduce (Van der Sluijs and Vaage, 2016).

The economic value of pollination services is calculated based on a huge assumption and needs a different approach that incorporates larger data (Melathopoulos et al. 2015). Kleijn et al. (2015) showed that 80% of crop pollination across five continents is carried out by just 2% of all wild bee species in the areas studied and contributes the vast majority of economic returns in agricultural systems. Besides ethical reasons, the authors argued that there are other important reasons for conserving other wild bees that do not pollinate crops and offer little economic return to farmers. For example, biodiversity benefits ecosystem services by providing insurance effects. Therefore, they argue that conservation of a wide range of bee species, not just those that are currently numerous on crops, is needed to maintain stable pollination services, as new bee species may become important pollinators in the future.

Pollinating services are provided by a wide range of animal species, mostly insects including honeybees, bumblebees, solitary bees, stingless bees, hover flies, butterflies, wasps, moths, beetles, midges, and other invertebrates, but also some vertebrates are known to pollinate such as bats, squirrels, parrots, hummingbirds, some primates, and humans (hand pollination) (Buchmann and Nabhan 1997; Klein et al. 2007). For agricultural crops, bees are the most important pollinators (UNEP 2010). In the past, most of the credit has been given to domestic honeybees. However, recent studies have shown that wild pollinators are more important contributors to global crop pollination than previously assumed (Breeze et al. 2011). Estimates for the UK indicate that managed honeybees (*Apis mellifera*) pollinate approximately no more than one third of the crops. Among the unmanaged pollinators, wild bees are important. Globally, over 25,000 species of bees are known (Chagnon et al. 2015). However, many insects other than bees are also efficient pollinators, providing 39% of visits to crop flowers (Rader et al. 2016). Wild insect pollinator species are regarded as the most effective pollinators of fruit crops (Chagnon et al. 2015). Klein et al. (2007) pointed to nine pollinator-dependent crops that did not exhibit proof of honeybee presence, and that three of these (atemoya, passion fruit, and vanilla) are now hand-pollinated in parts of the world, due in part to the reduced presence of the relevant wild pollinators.

Kleijn et al. (2015) showed that 80% of crop pollination across five continents is carried out by just 2% of all wild bee species in the areas they studied and contribute the vast majority of economic returns in agricultural systems. Besides ethical reasons, the authors argued that there are other important reasons for conserving other wild bees that do not pollinate crops and offer little economic return to farmers. For example, biodiversity benefits ecosystem services by providing insurance effects. Therefore, they argue that conservation of a wide range of bee species, not just those that are currently numerous on crops, is needed to maintain stable pollination services, as new bee species may become important pollinators in the future.

Crop yield and/or quality depend on both the abundance and diversity of pollinators (EASAC 2015). In general, a diverse community of pollinators provides more effective and stable crop pollination than any single species. Even when managed honey bees are present in high abundance, diversity of wild pollinators still improves crop pollination. Overall, the contribution of wild pollinators to crop production is undervalued (IPBES 2016b).

The impact of neonicotinoids on pollination has primarily been studied for oil seed rape agro-ecosystems. This crop is indeed especially relevant at least in the European context because, as stated by Budge et al. (2015): “Nowhere is this tension more evident than in the system we describe here with the world’s most widely used insecticide, the world’s most widely used managed pollinator and Europe’s most widely grown mass flowering crop.” These authors used combined data on large-scale pesticide use, yields of oliseed rape and honeybee colony losses over an 11 year period in England and Wales to study the impact of the three major neonicotioids used in that crop (imidacloprid, clothianidin, and thiamethoxam). They found that seed coating with imidacloprid had positive effects on yields in some years but negative effects on other years. Similarly, costs/benefits for farmers changed from year-to-year, prompting the authors to state: “Risk assessments assuming total control in the presence of a seed coating versus apocalyptic yield losses in their absence are simplistic and perhaps over-state the benefits”. At the same time, a correlation between honeybee colony losses and national-scale imidacloprid usage patterns was apparent and could not be ignored (Budge et al. 2015).

A field study conducted in southern England showed that neonicotinoids used as seed dressing for autumn-sown crops (oil seed rape (OSR) and winter wheat) contaminated adjacent soil and wildflowers (Botias et al. 2015). The pollen and nectar of these flowers were contaminated by thiamethoxam, clothianidin, imidacloprid, and thiacloprid (up to a frequency of 58.8%) in the following growing season with frequencies and concentrations declining between spring and summer. Concentrations were quite variable among samples (maximum concentrations: 19.12 ng/g for clothianidin in field margin soil and 28.6 ng/g in ORS cropland soil and 86.02 ng/g for thiamethoxam in hand collected wildflower pollen from OSG field margins and 25.55 ng/g for imidacloprid in bee-collected pollen during OSR blooms), suggesting high spatial heterogeneity in neonicotinoid accumulation and/or degradation. Frequency of occurrence and average concentrations were higher in pollen than in nectar. The authors estimated that 97% of all neonicotinoids entering the hive in pollen originated from wildflowers and only 3% from oil see rape. And important implication of this study is that it may explain why no impact of neonicotinoid on bees could be observed when comparing treated and non-treated fields because in both cases the bees were collecting most of the pollen and nectar from contaminated wildflowers, and very little from the treated crop. This implies that the impact of neonicotinoids on pollinators and hence on pollination service may have been underestimated and that experimental field studies are not good models for understanding the extent of the impacts. Another study in the USA reported similar findings (Long and Krupke 2016).

In a follow-up study, the authors further explored the contamination of neonicotinoid-treated OSR and field margin wild plants by neonicotinoids (Botías et al. 2016). The range of neonicotinoid concentrations was high in field margin wild plants (up to 106 ng/g for thiamethoxam for *Cirsium vulgare*) Maximum, but not average neonicotinoid concentrations were higher in field margin wild plants than OSR. As *C. vulgare* is notoriously attractive to many pollinators and its seeds eaten by different finch species, the high neonicotinoid concentration reported in this species, is of concern. These results may indicate that hedgerows and field margins, which are often managed to for biodiversity conservation and act as reservoirs for pollinators and antagonists of agricultural pests, may fail to deliver these functions (i.e., ecosystem services) if contaminated by neonicotinoids.

David et al. (2016) analyzed the content of neonicotinoids and fungicides in pollen from OSR and OSR field margin wildflowers and pollen collected by honey bees. Frequency of occurrence and mean concentrations of neonicotinoids was higher in OSR, lower in wildflowers and lowest in honey bee pollen. Pesticides concentrations were higher during ORS bloom than after. However, the highest single measured concentration for thiamethoxam (21 ng/g) was from a wildflower pollen sample (*Matricaria recutita*). The pesticide content of bumblebees and bumblebee pollen from rural and urban areas was also compared. Frequency of occurrence and mean concentrations of neonicotinoids were higher in rural samples. Frequency of occurrence and mean concentrations of neonicotinoids were higher in pollen from bumblebees as compared to pollen from honey bees, both collected during OSR bloom, presumably because bumblebees have a higher propensity to collect ORS pollen and forage over shorter distance, mostly within the neonicotinoid-contaminated area (David et al. 2016).

Sublethal concentrations of neonicotinoids were shown to alter the foraging behavior of bumblebees including a change in flower preference (bees exposed to thiamethoxam visiting more the larger, yellow flowers of *Lotus corniculatus* than the smaller, white flowers of Trifolium repens), an increase in the proportion of bees foraging, and an increase in the proportion of bees collecting pollen. Bees exposed to thiamethoxam learned to manipulate flowers faster but after more visits (Stanley and Raine 2016). These results suggest that exposure of bees to sublethal concentrations of neonicotinoids may alter their function as pollinators, possibly favoring some plant species over others, with potential implications for both crop production and wildflower reproductive success.

Widespread contamination of wild plants at field margins of arable crops has been demonstrated in other studies (Krupke et al. 2017). As a consequence, detrimental effects on survival of managed bees (Mogren and Lundgren 2016) as well as wild bees (Mallinger et al. 2015; Stanley and Raine 2016; Woodcock et al. 2016) have been documented. More recently, a correlation study between the use of pesticides, including neonicotinoids, and the abundance of butterflies in California has shown that the latter pesticides could be responsible for the decline of several species since 1996, when they were introduced in that State (Forister et al. 2016).

There is enough mechanistic understanding to put the question of causality beyond reasonable doubt. The detrimental effects on pollinators from the present scale of use of neonicotinoids are likely to impact pollination services, and in turn pollinator-dependent crop production.

### Impacts on natural systems for pest and weed control

Detrimental effects of systemic insecticides on natural predators and parasitoids have been discussed above, but most of the evidence comes from laboratory studies. Here are shown further mesocosms or field studies related to the dynamics of pest control in agricultural systems.

In cotton fields, both thiamethoxam and imidacloprid, applied as seed treatment, reduced abundance of beneficial arthropods, although in the case of imidacloprid only with applications at higher than recommended doses. However, it is important to note that sublethal effects were not evaluated in the study (Saeed et al. 2016). Uhl et al. (2015) studied trophic interactions in a three-level food chain mesocosm: wild strawberry *Fragaria vesca*, wood cricket *Nemobius sylvestris*, and nursery web spider *Pisaura mirabilis*. They found that a low imidacloprid rate (0.24 g/m^2^) reduced mass gain in crickets, whereas a high rate (2.4 g/m^2^) reduced feeding, mass gain, thorax growth, and mobility in crickets compared to the control. Both herbivory of crickets and predation by the spider were reduced at sublethal imidacloprid concentrations, with survival of crickets being higher in the low treatment. The experiment suggests that trophic interactions can be hampered even at sublethal concentrations of the insecticide (Uhl et al. 2016).

Secondary poisoning by preying on neonicotinoid-contaminated prey was already known from laboratory studies (Walker et al. 2007; Wanumen et al. 2016b), but it has now been confirmed under field situations: after feeding on contaminated aphid prey in wheat fields treated with thiamethoxam, the ladybird *Coleomegilla maculata* experienced sublethal effects that significantly impaired its predation capabilities (Bredeson et al. 2015). As a consequence, the target pest is not effectively eliminated by its natural enemy, potentially leading to pest resurgence. Surge of secondary pests may also occur when there is differential toxicity among two or more pest species. For example, where leaffolder (*Cnaphalocrocis medinalis*) incidence occurs along with planthoppers (*Nilaparvata lugens*) in rice fields of India, the use of neonicotinoids has resulted in increases of the leaffolder population. Stimulated fecundity of the leaffolder bugs on neonicotinoid-sprayed plants, coupled with reduced larval duration and low egg toxicity, were the major factors contributing to the upsurge of leaffolders (Chintalapati et al. 2016).

Overall, the combined effect of lethality on non-target natural enemies, secondary poisoning and sublethal impairments on their predatory ability results in deficient pest control and often leads to the resurgence of pests—a clear case of pest control failure (Kurwadkar and Evans 2016).

Indirect effects of pesticide seed treatments have been observed in regard to weed control. Thus, in a 2-year field study in the USA, the abundance of natural enemies (e.g., soil-dwelling seed predators and pathogens) that damage or destroy seeds of weeds in the agricultural soil was reduced in maize and soybean crops treated with pesticide-coated seeds (presumably a neonicotinoid and fungicides). Therefore, the weed seed banks in seed-treated plots would be larger and less diverse than those in untreated plots; hence, weed populations may increase in crops using pesticide seed treatments (Smith et al. 2016).

### Impacts on aquatic ecosystems

The contamination of agricultural soil with neonicotinoid and fipronil residues results mainly from the widespread use of pesticide-coated seeds (Douglas and Tooker 2015; Hladik et al. 2014). Eventually, soil residues move into the aquatic ecosystems, either by percolation and leaching through the soil profile (de Perre et al. 2015; Wettstein et al. 2016) or in surface runoff after rainfall and storms (Chrétien et al. 2017). Foliar sprays and drenches applied to orchard trees also contribute to the contamination of waterways (Englert et al. 2017; Kreutzweiser et al. 2008b). There is sufficient evidence to date to state that water borne residues of neonicotinoids and fipronil are currently impacting on aquatic ecosystems, as shown in the recent review by Sánchez-Bayo et al. (2016).

The main impacts include altering the invertebrate communities, mainly insects, that live in streams and ponds, which are responsible for the recycling of organic matter that falls into them (Englert et al. 2017). The main taxa affected have been discussed above and include key species such as midges and other Diptera such as mayflies, stoneflies, and caddisflies as well as predatory dragonflies, damselflies, bugs, and beetles. The most sensitive species are detritivorous that fulfill an essential role in the recycling of organic matter in streams (Kreutzweiser et al. 2008a), and low water residue levels induces sublethal effects that impair their ability to perform this role (Kattwinkel et al. 2016), while their elimination translates into alterations in ecosystem functions, like leaf litter breakdown (Englert et al. 2012). Most of these taxa have aquatic larval stages and produce a large biomass that feeds a diverse array of insectivorous vertebrates such as newts, frogs, lizards, aquatic shrews, and birds. The depletion of this food source can be quantified in mesocosm studies, as described in (Sánchez-Bayo et al. 2016a). New studies on experimental rice mesocosms have shown that while standard application of imidacloprid to rice seedlings reduces the abundance of dragonflies and water predatory bugs, dinotefuran did not produce any significant effects on the insect communities, and rather increased the abundance of midges and one species of dragonfly through indirect competition with other species (Kobashi et al. 2017).

The insect fauna in Germany over the past 20 years has recorded a drop in abundance of flying insects, mainly Diptera, of over 75% (Sorg et al. 2013). As many of these insects have aquatic life cycles, their disappearance is probably due to their larvae not having survived their aquatic phase. The consequences go beyond the realm of the aquatic ecosystem, since insects are the staple food source of many songbirds. The study by Hallmann et al. 2014 (see part B here) showed that in regions of the Netherlands where waterway contamination with imidacloprid was above 20 ng/L (ppt), populations of 14 species of birds have steadily declined in the past 20 years (Hallmann et al. 2014).

Risks of neonicotinoids to aquatic ecosystems are often been dismissed after some studies showed that current residue concentrations in water are not likely to be toxic (e.g., acute toxicity) to the common surrogate species used in regulatory risk assessment of chemicals. For example, clothianidin was detected at low concentrations in soil and water throughout a 2-year corn and soybean rotation. No short-term environmental risk was expected for the species investigated (*Daphnia magna*, *Hyalella azteca*, *Chironomus dilutus*, *Pimephales promelas*, and *Eisenia fetida*) because, with the exception of the amphipod, all species are very tolerant to this and other neonicotinoids (de Perre et al. 2015). This and similar studies funded by the chemical industry (Aslund et al. 2017) do not take into account the chronic lethality to sensitive aquatic organisms explained above, which is the real cause of concern for the long-term impacts on this ecosystem.

Aquatic vertebrates such as fish, however, may not be affected directly by residues of neonicotinoids in water. Treatment of oyster beds with imidacloprid to avoid burrowing shrimps after harvest did not appear to threaten populations of the endangered green sturgeon (*Acipenser medirostris*) in the west coast of the USA (Frew and Grue 2015; Frew et al. 2015). Nevertheless, the authors only compared the residual concentrations of imidacloprid in the water column (28 ppb) and shrimp (31 ppb) to the acute and chronic toxicity of this insecticide to a surrogate fish species (rainbow trout), since the actual toxicity to the sturgeon is unknown. It is likely that the risks of such contamination to a large fish like the sturgeon are negligible, even though sublethal effects cannot be ruled out (see Section B). Impacts on fish species, if any, would occur through indirect starvation after the invertebrate food source is depleted.

## Conclusions

**Fig.3:**
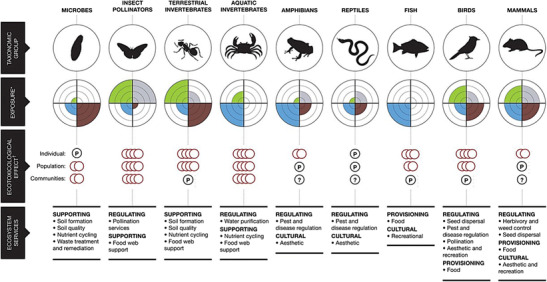
Summary of exposure routes and effects of neonicotinoids and fipronil on taxonomic groups. Routes of exposure are depicted by a quarter of circle for air (gray), plants (green), soil (brown), and water (blue). Exposure is scaled with five levels, and colored as variable circular sectors (empty: no route of exposure; small: potential route of exposure assumed neglectible; medium: relevant route of low exposure; large: relevant route of moderate exposure; extra-large: relevant route of high exposure). Ecotoxicological effects are scaled with four levels, according to the number (*n*) of imbricated red circles (*n* = 1: potential effects assumed neglectible under normal exposure conditions; *n* = 2: evidence of effects which can occur at high doses or after chronic exposure; *n* = 3: evidence of effects which can occur at moderate doses; *n* = 4: evidence of effects which can occur at low doses or after acute exposure). Probable effects are reported with Ⓟ when no accurate judgment could be made due to incomplete evidence, but for which data suggest a potential effect that can occur at high doses or after chronic exposure. A question mark is reported in situation where no assessment could be made because of lack of evidence (e.g, no data available). Major ecosystem services regulated and supported by these taxonomic groups are listed at the bottom

In the past 3 years, we have gained a greater description about the exposure to neonicotinoids and fipronil and a greater understanding about their effects in arthropods and other species, as a result of a large research effort made worldwide. Fipronil has been studied to a lesser extent. A summary of our worldwide integrated assessment for taxonomic groups is synthesized in Fig. 3, with the exposure data to these systemic insecticides (Giorio et al., 2017, this special issue), their ecotoxicological effects and the related ecosystem services.

Research on bees has revealed new aspects of sublethal effects, including the reduced fecundity of queen bees, impairment of sperm in drones, negative interactions with parasites and the immune system. Our knowledge of acute toxicity has also broadened to include some wild bee species, while the mixture toxicity in combination with other pesticides or infectious agents has reported some synergisms that are more pronounced than simply additive. Impacts of neonicotinoids and fipronil at the population level of bumblebees were known to some extent, but have now been compared among countries with different environments. The impacts on other wild bees were unknown and recent studies have shown that they are more sensitive to neonicotinoids than the honey bee. These impacts on pollinators are a real cause for concern, with a few studies showing a correlation between the use of neonicotinoids and the declines in wild bees and butterflies in Europe and America. Their presence in hedgerows or field margins may expose pollinators to drift or uptake of active molecule by growing plants.

It was known that neonicotinoids were very toxic to natural enemies of agricultural pests and caused secondary poisoning. The new information gathered in this area has only contributed to enlarge the range of species tested, whether predatory or parasitoids, thus confirming the unsuitability of neonicotinoids for integrated pest control programs.

Little progress has been made, however, in evaluating the effects of these insecticides on soil organisms, with the exception of a study showing the possible bioaccumulation of fipronil in earthworms. Some field studies on indirect impacts on non-target termites and other arthropods have reported ambiguous findings.

New studies on aquatic invertebrates have focused on acute and chronic toxicity of clothianidin, thiamethoxam, and a few new compounds to a wider range of taxa, since the data available until recently referred almost exclusively to imidacloprid. There is now sufficient data to set protective limits for aquatic invertebrate communities in the legislation. Some studies have shown how the current contamination of surface waters in many countries are effectively damaging the underlying insect communities that provide a rich food source not only to fish but also birds and other insectivorous vertebrates. The chronic exposure to low levels of neonicotinoid residues in water are causing a long-term lethality in most aquatic species, which eliminates entire populations form the affected areas.

Fipronil and the neonicotinoids imidacloprid and clothianidin exert a wide range of deleterious sublethal neurological effects in terrestrial vertebrates, such as rats and bats. Ingestion of treated seeds by birds can cause sublethal effects on immunity and can be lethal at times. Aquatic vertebrates, however, are very unlikely to be exposed to lethal or sublethal concentrations of neonicotinoids in their natural environment, whereas environmental concentrations of fipronil may be sufficiently high to harm fish.

Overall, the negative impacts of neonicotinoids and fipronil on terrestrial and aquatic invertebrates are translated in indirect impact for the entire ecosystems. Invertebrates constitute the main food source for an innumerable number of insectivorous vertebrates and fulfill an essential role in recycling organic matter in the soil as well as in water. The consequences of losing the invertebrate fauna due to continuous exposure to ubiquitous residues of neonicotinoids and fipronil are thus far reaching and cannot be ignored any longer.
